# Systematic Analysis of Alternative Splicing in Transcriptomes of Multiple Sclerosis Patient Brain Samples

**DOI:** 10.3390/ijms26178195

**Published:** 2025-08-23

**Authors:** Müge Sak, Julia H. Chariker, Eric C. Rouchka

**Affiliations:** 1 Kentucky IDeA Networks of Biomedical Research Excellence Data Science Core, Department of Neuroscience Training, University of Louisville, Louisville, KY 40292, USA; muge.sak@louisville.edu (M.S.); julia.chariker@louisville.edu (J.H.C.); 2 Kentucky IDeA Networks of Biomedical Research Excellence Data Science Core, Department of Biochemistry and Molecular Genetics, University of Louisville, Louisville, KY 40292, USA

**Keywords:** multiple sclerosis, RNA-seq, differential expression, alternative splicing

## Abstract

Multiple sclerosis (MS) is an autoimmune and neurodegenerative disease affecting approximately 1 million people in the United States. Despite extensive research into the mechanisms of disease development, many aspects of the biological changes during MS progression and the varying symptoms among patients remain unclear. In the era of high-throughput sequencing, transcriptome databases are flooded with data. However, bulk RNA sequencing (RNA-seq) data are typically used only for differential gene expression analysis. Alternative splicing, a key process that alters the transcriptome, can also be identified from bulk data. Here, we accessed 11 studies with bulk RNA-seq data of postmortem MS patients’ brain samples via NCBI’s Gene Expression Omnibus (GEO). We extracted additional information from these data by identifying exclusively alternatively spliced genes via replicate multivariate analysis of transcript splicing (rMATS) analysis. Our analyses revealed that changes in RNA splicing mediate distinct biological signals compared to those driven by differential gene expression. Gene ontology and protein do-main analyses of genes exclusively regulated by alternative splicing revealed distinct molecular differences between progressive and relapsing–remitting MS as well as among lesions from different brain regions and between white and gray matter. These findings highlight the critical role of alternative splicing and its associated pathways in MS disease development and progression.

## 1. Introduction

Alternative splicing is a major mechanism that plays an important role in transcriptome and proteome diversity [1] with roughly 15% of genetic diseases and cancers associated with alternative splicing [2]. While transcriptomic studies have been widely used to elucidate differentially expressed genes, less emphasis has been placed on identifying alternative splicing within these same datasets. However, alternative splicing may yield important biological insights not found in the differentially expressed genes. As a case in point, a recent study of publicly available datasets across 199 comparisons found on average 4327 genes were significantly differentially expressed and 2247 genes were significantly alternatively spliced. Among these, on average, 1252 genes (33.6%) of the differentially expressed genes were also differentially spliced [3]. A second study similarly investigated the alternative splicing events and found that roughly 22% of the differentially spliced genes were also differentially expressed, leaving 78% of the alternatively spliced events to be unexplored [4]. The conclusion of both of these studies is that bulk-RNA sequencing differential gene expression analysis alone overlooks the importance of alternative splicing events [3,5].

Multiple sclerosis (MS) is an autoimmune neurodegenerative disease of the central nervous system (CNS) that affects approximately 1 million people in the United States [6], 2.9 million people worldwide, and the estimated MS prevalence in Europe was calculated at 142.8 per 100,000 individuals [7]. Although MS is not typically classified as an inherited disease, 233 MS risk loci have been identified in genome-wide association studies [8]. Among other possibilities, these variants have the potential to affect gene regulation and splicing mechanisms. Putscher et. al. showed MS risk variants are associated with alternative splicing in C-Type Lectin Domain Containing 16A (*CLEC16A*), EF-Hand Calcium Binding Domain 13 (*EFCAB13*), Gasdermin B (*GSDMB*), Major Histocompatibility Complex, Class I, C (*HLA-C*), Interleukin 7 Receptor (*IL7R*), Non-SMC Condensin II Complex Subunit H2 (*NCAPH2*), SP140 Nuclear Body Protein (*SP140*), and Ts Translation Elongation Factor, Mitochondrial (*TSFM*) genes [9]. Additionally, a recent study showed that alternative splicing events are common and occur independently of differential expression in MS [10].

MS has been categorized into three main types: relapsing–remitting MS (RRMS), secondary progressive MS (SPMS), and primary progressive MS (PPMS). RRMS patients experience distinct periods of symptoms, known as attacks, that are followed by remission periods. Patients with the progressive forms of MS (PPMS and SPMS) go through a continuous progression of disease, leading to disability. Some patients may experience an initial stage of RRMS that develops into the progressive type of MS (SPMS), while some patients are directly diagnosed with progressive MS (PPMS) [11]. In addition to the environmental factors for the risk of MS such as obesity, vitamin D deficiency, and smoking, the epidemiologic studies show that the northern European descendants carry the highest risk for MS diagnosis [12].

Here, we aimed to unravel alternative splicing events in MS by reanalyzing 11 studies with the bulk RNA-seq data of postmortem MS patients’ brain samples from GEO [13]. The sample sites were mainly from European countries representing the highest prevalence of MS diagnosis in those countries [12] (Table 1). We identified 25 different comparisons from these studies (Table 2). In most comparisons, fewer than 10% of the genes affected by differential expression or alternative splicing were both differentially expressed and alternatively spliced (Table 3). This suggests while there are shared biological signals that were affected by both differential expression and alternative splicing, there are also distinct pathways altered by only alternative splicing events. Our study uncovered the changes specifically driven by alternative splicing events in the MS brain by analyzing all of the publicly available RNA-seq datasets from previous studies. Our approach addressed a critical gap in our understanding of alternatively spliced genes and their roles in MS pathogenesis.

## 2. Results

### 2.1. Differentially Expressed and Alternatively Spliced Genes Showed Low Percentages of Overlap and High Variety in Enriched Pathways

**Figure 1:**
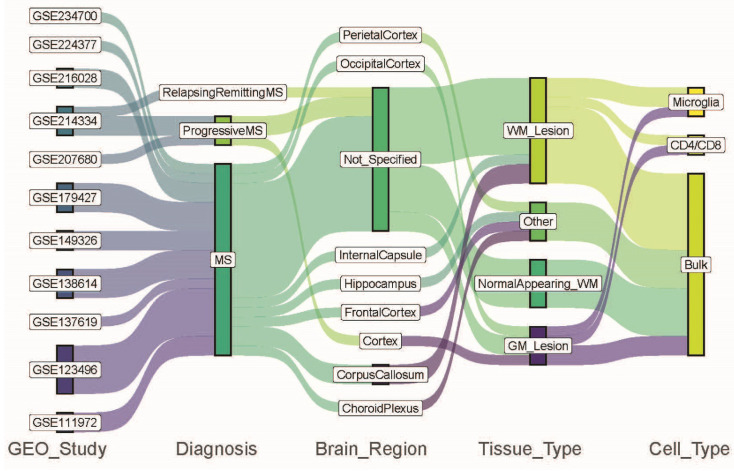
Data characteristics for each dataset from the metadata files and identified four different comparison categories: diagnosis, brain region, tissue type, and cell type. The colors trace how data from various GEO studies flow through the different comparison categories like diagnosis, brain region, tissue type, and cell type.

We identified the data characteristics for each dataset from the metadata files and determined four different comparison categories: diagnosis, brain region, tissue type, and cell type (Figure 1, Table 2). We had twenty-five comparisons across the eleven studies according to the diagnosis, brain region, tissue type and cell type information (Table 2). We determined the differentially expressed genes (DEGs) and alternative splicing events (ASEs) in each comparison as well as only DEGs, only ASEs, and the intersection between the two (Table 3). We found that the overlap of DEGs and ASEs ranged from 0% to 16.03% of the total of DEGs and ASEs with an average of 2.19%. In seven comparisons, while there were fewer than 100 DEGs, there were several hundred ASEs (C5, C6, C7, C8, C18, C21, and C22) (Table 3). On the other hand, there was only one study with the converse results where there were three ASEs in GSE234700, while there were over 1000 DEGs (C25). Two comparisons from GSE149326 had fewer than 100 genes in both categories (C14, C15) (FDR < 0.05) (Table 3).

The enriched gene ontology biological pathways (GO:BP) [25] for DEGs, ASEs, and the intersects for each comparison are shown in Figure S1. We found that in the GSE207680, GSE123496, and GSE214334 studies, while DEGs were enriched in transport, localization, synaptic signaling, and immune system-related pathways, ASEs were enriched in nervous system development, neurogenesis, and neuron differentiation-related pathways (Figure S1). In the study GSE111972, the white matter comparison showed that ASE and DEG genes were both enriched in immune system processes, but only the genes that were both ASE and DEG were enriched in Major Histocompatibility Complex (MHC) protein complex assembly, which plays a crucial role in MS development and progression [26]. Similarly, in the GSE138614 study, the genes that were both ASEs and DEGs were enriched in nervous system-related pathways (Figure S1).

We performed Pearson statistics for *p*-values of DEGs and ASEs for each comparison. Correlation plots and the correlation coefficients did not support any correlation between differential expressions and alternative splicing events (Figure S2).

These findings indicate the importance of the analysis of ASEs, as the overlap with DEGs is very limited. We found ASEs were enriched in important MS-related pathways that did not show up in enrichment analysis for DEGs. Our further analysis focused on only the ASEs in each comparison and the identification of overlaps from different studies.

### 2.2. Diagnosis Comparisons

We had three comparisons from two studies for progressive MS (PMS) and one comparison for relapsing–remitting MS (RRMS) samples (Table 4). The remaining studies were labeled as MS diagnosis. A comparison of all MS lesions vs. non-MS samples (Table 4) showed RNA-binding motif 29 (*RBM39*) as the only common exclusively alternatively spliced gene. Serine/arginine-rich splicing factor 5 (*SRSF5*) was common in fourteen out of fifteen comparisons and the Arginine and Serine Rich Protein 1 (*RSRP1*), Ribosomal Protein S9 (*RPS9*), Erythrocyte Membrane Protein Band 4.1 Like 2 (*EPB41L2*), Heterogeneous Nuclear Ribonucleoprotein H1 (*HNRNPH1*), Heat Shock Protein Family A Member 9 (*HSPA9*), DEAD-Box Helicase 3 X-Linked (*DDX3X*), Prostaglandin E Synthase 3 (*PTGES3*), Cysteinyl-TRNA Synthetase 1 (*CARS1*), SWI/SNF Related, Matrix Associated, Actin Dependent Regulator Of Chromatin Subfamily C Member 2 (*SMARCC2*), A-Kinase Anchoring Protein 8 Like (*AKAP8L*), Mitochondrial Carrier 1 (*MTCH1*), Eukaryotic Translation Initiation Factor 4 Gamma 2 (*EIF4G2*), and WW Domain Containing Adaptor With Coiled-Coil (*WAC*) genes were alternatively spliced but not differentially expressed in thirteen out of fifteen comparisons. These genes are commonly involved in cellular functions, chromatin remodeling, and RNA splicing, which can affect MS development and progression [26,27,28,29].

**Figure 2:**
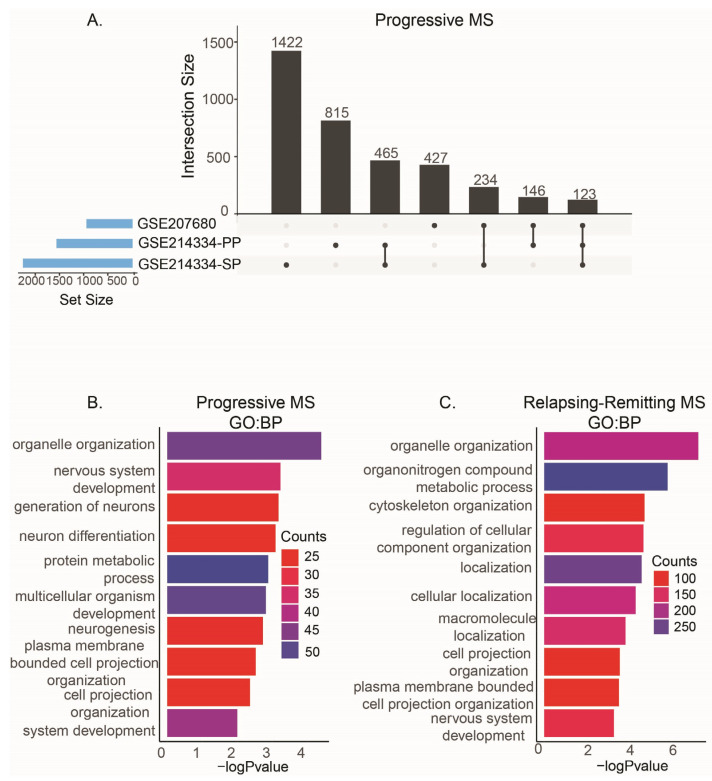
Diagnosis comparisons. (**A**). Upset graph showing the overlaps of alternatively spliced genes in each comparison of PMS. PP: primary progressive, SP: secondary progressive. (**B**). Enriched GO:BP pathways for genes commonly alternatively spliced in all three PMS comparisons. (**C**). Enriched GO:BP pathways for genes alternatively spliced in RRMS.

While PMS involves steady and gradual functional decline either from the onset of the disease (primary-progressive MS) or following an initial relapsing–remitting phase (secondary-progressive MS), RRMS patients experience relapsing and remitting phases of their symptoms [30]. Our PMS comparisons had 123 common alternatively spliced genes (Figure 2A, Table S1). These genes were mainly enriched in organelle organization, nervous system development, neuron generation and differentiation, and neurogenesis (Figure 2B). We had one comparison for RRMS patients in which 1038 genes were alternatively spliced but not differentially expressed. These genes were enriched in organelle organization and cellular localization pathways (Figure 2C).

### 2.3. Brain Region Comparisons

**Figure 3:**
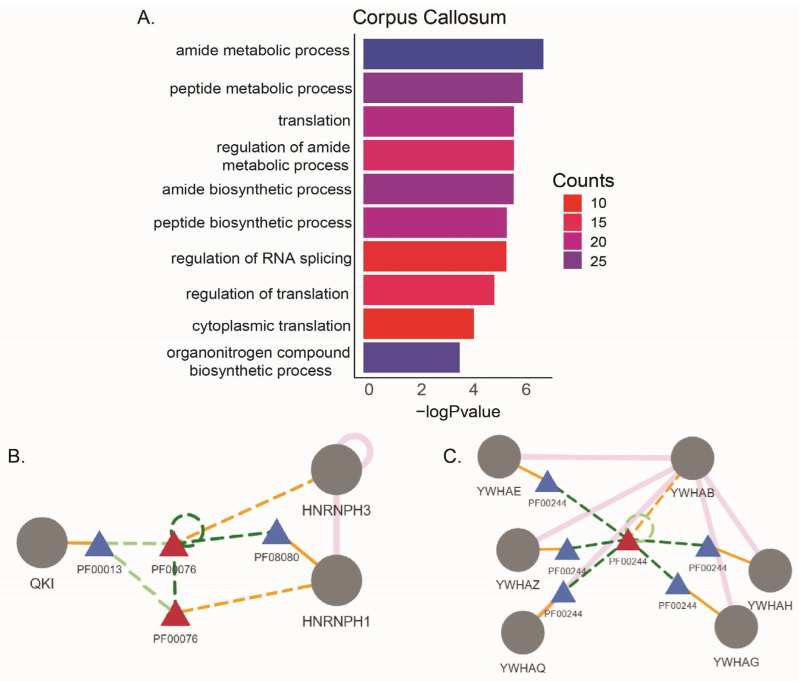
(**A**) Enriched GO:BP pathways for ASEs in CC tissue lesions from two studies. DIGGER schemas for (**B**) HNRNPH1 and HNRNPH3 interacting domains with QKI. (**C**) Protein 14-3-3 subunits interacting domains. Blue triangle: domain node. Red triangle: missing node. Pink line: edge from protein-protein interaction. Solid orange line: protein domain node. Orange dotted line: missing protein domain. Dark green dotted line: edge of missing domain. Light green dotted lines: edge of missing domain (predicted).

Abnormality in corpus callosum (CC) is observed in most MS cases [31]. We compared the alternatively spliced but not differentially expressed genes from CC lesions in two studies (Table 5). We found 114 genes were commonly alternatively spliced. These genes were mostly enriched in amide and peptide metabolic processes (Figure 3A). We found in these two studies 36 identical alternative splicing events (including the coordinates) in 34 genes (Table 6 and Table S2). These genes include Bridging Integrator 1 (BIN1), which is localized with Myelin Basic Protein (MBP) in CC and associated with myelination in developmental process. The differential expression of BIN1 isoforms was observed in the brains of Alzheimer’s disease patients, and the loss of BIN1 parallels myelin loss in multiple sclerosis brain lesions [32]. A KH domain containing RNA binding protein (QKI) has isoforms directly associated with hypomyelination [33], and Secreted Phosphoprotein 1 (SPP1/OSP) is found upregulated in MS lesions [22]. We further looked into the downstream effects of these alternative splicing events using NEASE [34] and found that nineteen of these events were predicted to result in changes in protein domains (Table S3). The skipped exon (SE) events in HNRNPH1 and HNRNPH3 genes are predicted to affect RNA recognition motif (PF00076), which can affect their interaction with QKI and RNA splicing (Figure 3B). Also, the ASE in the 14-3-3 protein beta subunit gene (YWHAB) is predicted to disrupt its structural domain (PF00244), which is required for its assembly with the other subunits of the protein (Figure 3C). The protein 14-3-3 is reported to be one of the severity markers for MS in cerebrospinal fluid (CSF) [35]. The other brain region comparisons were from one study, and we identified that the alternatively spliced genes were enriched in various metabolic, organization, and localization pathways in MS lesions from cortex, frontal cortex, occipital cortex, parietal cortex, hippocampus, choroid plexus, and internal capsule (Figure S3).

### 2.4. Tissue Type Comparisons

**Figure 4:**
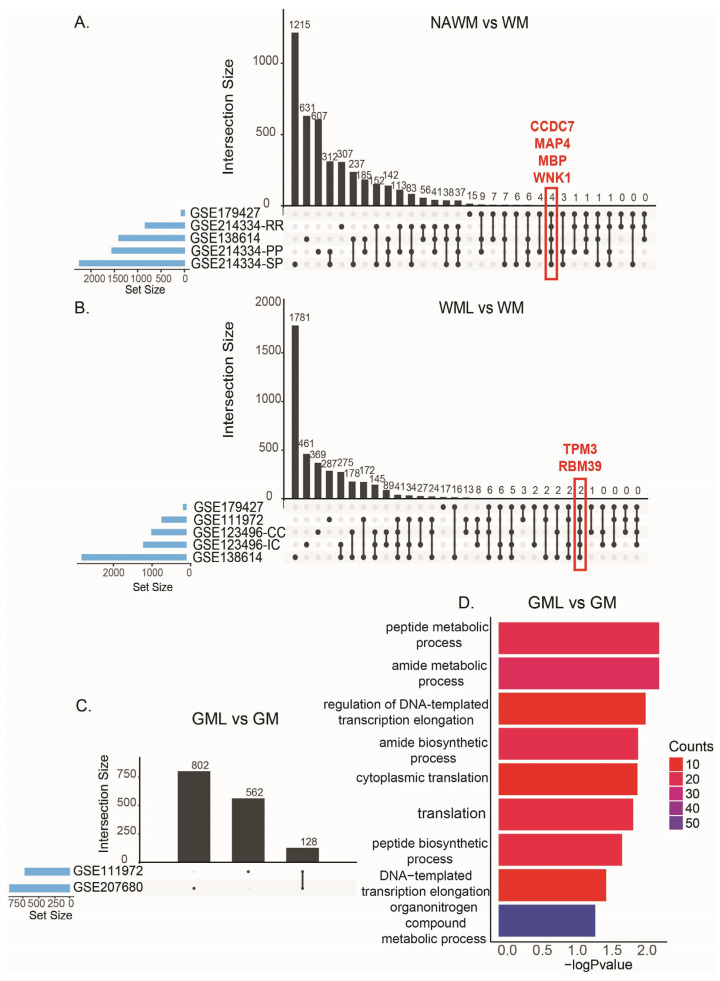
Tissue type comparisons. (**A**): Upset graph showing the overlaps of alternatively spliced genes in each comparison of NAWM to WM. RR: relapsing–remitting, PP: primary progressive, SP: secondary progressive. The genes that were common in all comparisons are shown in red. (**B**): Upset graph showing the overlaps of alternatively spliced genes in each comparison of WMLs to WM. CC: corpus callosum, IC: internal cortex. The genes that were common in all comparisons are shown in red. (**C**): Upset graph showing the overlaps of alternatively spliced genes in each comparison of GML to GM. (**D**): Enriched GO:BP pathways for genes commonly alternatively spliced in GML vs. GM comparisons.

Five comparisons were made from three different studies comparing NAWM and WM tissues (Table 7). We found that Coiled-Coil Domain Containing 7 (*CCDC7*), Microtubule-Associated Protein 4 (*MAP4*), Myelin Basic Protein (*MBP*), and WNK Lysine Deficient Protein Kinase 1 (*WNK1*) were commonly alternatively spliced in all comparisons (Figure 4A). MBP is the main component of myelin sheath and is widely studied in MS [36]. *CCDC7* has a high frequency of SNPs in myalgic encephalomyelitis/chronic fatigue syndrome [37]. MAP4 polymerizes with other microtubule-associated proteins and regulates the properties of microtubules, which are strongly associated with neurodegenerative diseases [38]. Also, a mutation on *WNK1* is found to cause a type of neuropathy [39].

For WMLs and WM, we had five comparisons from four different studies. Tropomyosin 3 (*TPM3*) and RNA binding motif 39 (*RBM39*) were commonly alternatively spliced in all comparisons (Figure 4B).

Two comparisons for GML vs. GM showed 128 common alternatively spliced genes (Figure 4C). These genes were enriched in peptide and amide metabolic processes and translation (Figure 4D). In these two comparisons, there were 52 identical ASEs between GML and GM tissues (Table S4). Further analysis of these identical ASEs showed 39 of them are predicted to affect the variety of domains of different proteins involved in lipid metabolism and cell signaling functions, including the protein 14-3-3 subunit YWHAB’s structural domain (Table S5).

### 2.5. Cell Type Comparisons

#### 2.5.1. Microglia

We had three comparisons from two studies that included microglial cells from MS patient brains (Table 8). We found the protein 14-3-3 epsilon subunit (*YWHAE*) and Ribosomal protein S9 (*RPS9*) genes were commonly alternatively spliced but not differentially expressed in microglial cells.

#### 2.5.2. CD4/CD8 T-Cells

We used transcriptome data from one study comparing differential transcriptomes of CD4 and CD8 T-cells in WMLs and GML to normal WM and GM to identify the alternatively spliced genes in these cells (Table 8). We found 125 genes were alternatively spliced in both WMLs and GMLs in CD4/CD8 T-cells. We found 109 identical alternative splicing events in 82 genes (Table 9 and Table S6).

**Figure 5:**
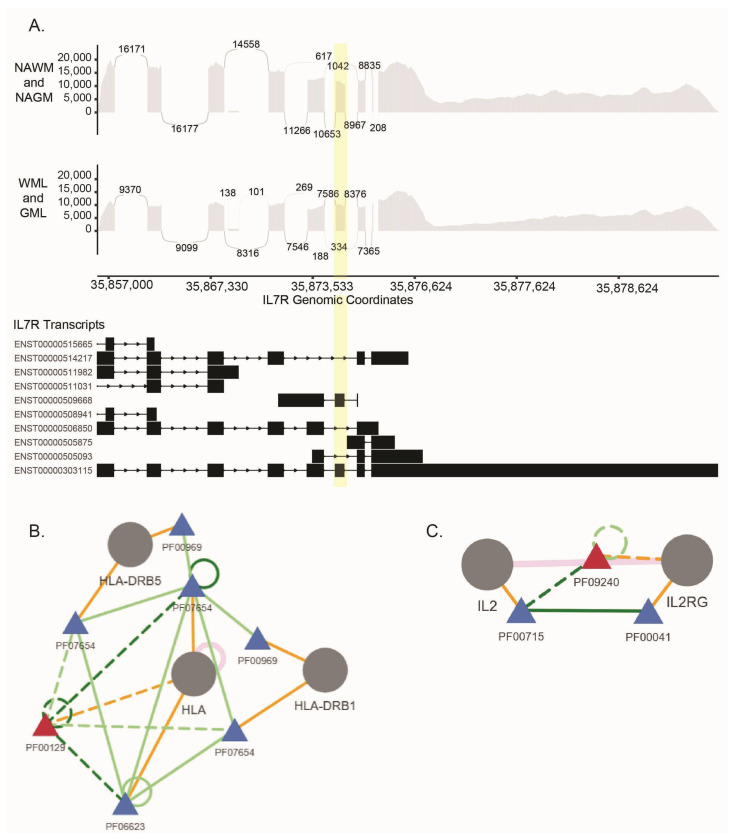
(**A**). Sashimi plot and known transcripts of the *IL7R* gene that are differentially spliced in WMLs and GMLs in CD4/CD8 T-cells. The numbers represent the sequence-based junction counts. The identified skipped exon 6 is highlighted in yellow. DIGGER schemas for (**B**). The disrupted interaction of HLA with HLA-DRB1 and HLADRB5 through its missing PF00129 domain. (**C**). The disrupted interaction of IL2RG and IL2 through its missing PF09240 domain. Blue triangle: domain node. Red triangle: missing node. Solid orange line: protein domain node. Orange dotted line: missing protein domain. Dark green dotted line: edge of missing domain. Light green dotted line: edge of missing domain (predicted). Pink line: edge from protein-protein interaction. Solid dark green line: edge from domain–domain interaction. Solid light green line: edge from domain–domain interaction (predicted).

Among these genes, HNRNPA1, HNRNPC, and HNRNPU are involved in RNA processing and have been linked to neurodegenerative diseases such as amyotrophic lateral sclerosis and frontotemporal dementia [40]. HLA-A and HLA-B are part of the human leukocyte antigen (HLA) complex, which plays a crucial role in the immune system. Variations in these genes are associated with an increased risk of MS [41]. Variations in *IL7R* (interleukin 7 receptor) are strongly linked to the risk of developing MS [42]. *IL7R* also has a risk allele shown to be associated with alternative splicing [10]. The exon 6 skipping in the transcript for the *IL7R* dependent on SNP rs6897932 is prominent [43]. The hg38p12 coordinates we found in the exon skipping event in *IL7R* (chr5:35,874,448-35,874,542) (Figure 5A, Table S6) overlaps with the exon 6 of *IL7R* and the location of the MS risk allele chr5:35874473. This finding strongly supports the risk allele-dependent alternative splicing event in the *IL7R* gene.

Our further analysis of predicting the effect of the alternative splicing events on protein domains resulted in 63 alternative splicing events predicted to affect the protein domains of 56 genes (Table S7). One of these is the HLA protein PF00129 domain required for interactions with HLA-DRB1 and HLA-DRB5 (Figure 5B), which have the strongest genetic associations with MS [41]. While the functional roles of HLA-DRs are complex, they are shown to contribute to MS through different mechanisms interactions with MS-associated infectious organisms and autoantigens [44].

We also found that an alternative splicing event in *IL2RG* may affect the PF09240 domain that disrupts its interaction with IL2 (Figure 5C). The IL2–IL2R pathway is involved in the differential induction of autoimmune responses and tolerance [45].

We conducted an additional analysis excluding the CD8+ T-cells and compared only the CD4+ T-cells in white matter and gray matter lesions. The results show additional 27 identical alternative splicing events in 25 genes (Table S8). These genes included *CCL5* (C-C Motif Chemokine Ligand 5), which is a chemokine that plays important roles in inflammatory diseases including MS [46]. CCL5 protein levels are high in MS patients and elevate as the disease progresses [47], which indicates this alternative splicing event in CD4+ T-cells may contribute to the MS pathogenesis. Additionally, *CD3D* (CD3 Delta Subunit of T-Cell Receptor Complex) plays a vital role in T-cell development, differentiation, and T-cell receptor signaling. It is suggested as a potential cause for MS pathogenesis [48] and a target gene for new drug development [49].

## 3. Discussion

Alternative splicing has been underexplored in bulk RNA-seq. Recent studies highlight the importance of identifying these genes and investigating their impact on transcriptome changes [3,4,5]. In our previous study, we utilized large-scale transcriptome data from postmortem MS patient brains (GSE138614) to delineate the gene expression and alternative splicing changes in different types of lesions. Our findings suggested alterations in splicing in lesions offer additional insights to understand the pathology of MS [5]. Here, we extended our previous work by performing comprehensive analyses on all publicly available bulk RNA-seq data of postmortem MS patients’ brain tissues to close the gap in the knowledge of alternatively spliced genes in MS by analyzing them independently of differential expressions. We downloaded raw sequencing files and employed differential expression and alternative splicing analysis for each study separately; therefore, we did not require any control for the batch effects. After the identification of differentially expressed and alternatively spliced genes in each study, we focused on only alternatively spliced but not differentially expressed genes for further analysis. We identified the common exclusively alternatively spliced genes in different studies with the same type of comparisons. Further downstream analysis of these ASEs allowed us to predict the disrupted protein domains and protein–protein interactions. While NEASE is a powerful tool to predict the downstream effects of alternative splicing, it does not take the specific alternative splicing event (SE, RI, MXE, A3SS, or A5SS) into account; therefore, results should be interpreted carefully.

Although it is known there are different types of MS with distinct characteristics for development and progression, the metadata from most of the studies did not provide diagnosis details. Therefore, there were significant sample size disparities in our diagnoses comparisons that may constrain the extent to which the results can be generalized. We found alternative splicing events in *RBM39* in all MS diagnosis comparisons. *RBM39* is mainly involved in RNA splicing, and its dysfunction can lead to degenerative diseases [50]. In the studies that provided the diagnosis for PMS and RRMS, we were able to identify distinct features. While RRMS samples had alternatively spliced genes involved in organelle organization and cellular localizations, the exclusively alternatively spliced genes in PMS samples were mainly enriched in neurogenesis and nervous system pathways. These genes included *MBP*, Abelson Helper Integration Site 1 (*AHI*), Ankyrin 3 (*ANK3*), and Ataxin 3 (*ATXN3*), which are associated with neuronal differentiation, function, and cell death [51,52,53], Immunoglobulin Mu DNA Binding Protein 2 (*IGHMBP2*) associated with spinal muscular atrophy [54], Phosphatidylinositol Binding Clathrin Assembly Protein (*PICALM*) associated with Alzheimer’s disease [55], SPG7 Matrix AAA Peptidase Subunit, Paraplegin (*SPG7*) associated with Spastic Paraplegia [56], Transcription Factor 4 (*TCF4*), involved in neurodevelopmental disorders [57], and Tetratricopeptide Repeat Domain 19 (*TTC19*), which carries mutations linked to neurodegeneration [58]. This suggests that PMS has distinct alternatively spliced genes that may be contributing to the worsening of the symptoms and progression in patients that may be potentially biomarkers for diagnosis and patient stratification, as well as novel drug targets for PMS management, halting the progression and treatment.

Disrupted lipid metabolism is one of the hallmarks of MS. However, how it influences disease processes remains uncertain [59]. The myelin protein breakdown and activation of remyelination is regulated by lipid metabolism. However, this process is often disrupted in MS. Various cell types in the central nervous system such as astrocytes and microglia influence the lipid metabolism, and new myelin sheaths are commonly generated by oligodendrocytes [60,61]. In our study, we found that the genes involved in lipid metabolism were exclusively alternatively spliced such as Acyl-CoA Dehydrogenase Very Long Chain (*ACADVL*), Ceramide Synthase 2 (*CERS2*), ELOVL Fatty Acid Elongase 5 (*ELOVL5*), and FIG4 Phosphoinositide 5-Phosphatase (*FIG4*), which can disrupt the regulation of fatty acids, sphingolipids, membrane and vesicle lipids impacting both the brain and the immune system [62,63]. Further research on the functional properties of the genes we identified and their alternatively spliced forms may greatly explain the lipid metabolism changes in MS and advance research in reactivating the remyelination mechanisms for demyelinated lesion repair.

Corpus callosum (CC) is the largest white matter tract, forming the connection between the two cerebral hemispheres. It is involved in the performance of complex tasks. In MS, the CC is frequently compromised in patients, and CC lesions are considered as sensitive and specific indicators of the disease. Studies suggest that the reduced CC integrity that accompanies MS plays a part in MS-related dysfunctions [64]. We identified 36 identical alternative splicing events (including the coordinates) in 34 genes specifically in CC from two studies. These genes included Alpha-2-Macroglobulin (*A2M*), which is involved in neuroprotection and has been studied as a potential biomarker for MS [65,66]. In addition, *BIN1* has differentially expressed isoforms in the brains of Alzheimer’s disease patients. Also, the loss of *BIN1* parallels myelin loss in multiple sclerosis brain lesions neurodegeneration [32], and *DDX5* is involved in *MBP* regulation [67]. The downstream effects of the alternative splicing events in *HNRNPH1* and *HNRNPH3* are predicted to affect their domains that interact with QKI. QKI is involved in oligodendrocyte differentiation, and the deletion of one of its isoforms in oligodendrocytes leads to severe CNS hypomyelination [33]. QKI-6 isoform is shown to act upstream of HNRNPH and regulate alternative splicing specifically in myelinating glia [68]. Our data showing a disrupted domain–domain interaction between HNRNPH and QKI may hint at a mechanism of dysregulation of myelination in MS.

When comparing NAWM to WM, we identified that *MBP*, the main component of the myelin sheath, was one of the genes that was alternatively spliced but not differentially expressed. The early studies for MS biomarker identification in CSF suggested MBP levels had a low prediction value for early diagnosis [69]. However, MBP levels in CSF were reported as a potential biomarker of disability progression in SPMS patients [70]. Also, in a recent study, the MBP content in oligodendrocyte-derived extracellular vesicles was found to be significantly high in MS patients [36]. Our results showing there are significant alternative splicing events in *MBP* in NAWM may lead to the identification of a specific isoform of MBP that could be a biomarker for early diagnosis.

The protein 14-3-3 is considered one of the disease severity markers in MS [71]. We identified alternative splicing events in the beta subunit gene (*YWHAB*) and epsilon subunit gene (*YWHAE*) of protein 14-3-3. Disruption of the structural domains of protein 14-3-3 subunits can lead to dysregulation in signal transduction and cell cycle regulation as well as innate immunity. *YWHAE* is abundant in the brain and involved in neurodevelopment and neural signaling. It has been identified as a biomarker for neurodegenerative diseases and is linked to conditions like Alzheimer’s disease and HIV-associated neurocognitive disorders [72]. *YWHAE* has splice variants that are protein coding P62258-1 and P62258-2. The latter shorter form of *YWHAE* cannot dimerize with the zeta subunit of the protein 14-3-3 (*YWHAZ*), and it results in Miller–Dieker syndrome, which is a rare neurodevelopmental disease that is associated with poor myelination and brain malformations [73]. Therefore, our data suggest that the disruption of protein 14-3-3 assembly in MS may be the result of the alternative splicing events in its subunit genes.

While CD4 T-cells are primarily involved in initiating and sustaining the inflammatory response that leads to demyelination in MS, CD8 T-cells are generally responsible for contributing to the immune attack as well as regulating the immune response [74]. In the study GSE216028, there were only two CD8 T-cells samples from GML [22]. Therefore, for our analysis, we combined the CD4 and CD8 T-cells for the purpose of having enough samples in each group of comparisons. We found 109 identical alternative splicing events in 82 exclusively alternatively spliced genes in these cells from both WMLs and GMLs. These genes included *IL2RG* and *IL32*, which are linked to autoimmune diseases [75], and *IL7R*, which is the most prominent example of a risk allele-associated alternatively spliced gene in MS [10,43]. It is shown that exon 6 skipping in the transcript for the *IL7R* is dependent on MS risk allele SNP rs6897932 [43]. The coordinates we found in this exon skipping event in *IL7R* (chr5:35,874,448-35,874,542) overlaps with exon 6 of *IL7R*. This is a strong indication that we were able to identify this specific risk allele-associated alternative splicing event from postmortem human brain tissues specifically in CD4/CD8 T cells from WMLs and GMLs. Furthermore, we predicted a possible disruption of HLA interactions with HLA-DRB1 and HLA-DRB5. HLA-DRB1*15:01 and HLA-DRB5*01:01 alleles are the most significant genetic risk factors for MS and always occur together because of their near-perfect linkage disequilibrium [44]. Also, the functional studies suggest the involvement of both in an antigen-presenting mechanism to T-cells in MS [76]. Functional analysis of the identified alternative splicing event in our study may unravel a novel mechanism of HLA interactions in MS.

Our analysis for only CD4 T-cells showed an additional 27 alternative splicing events in white matter and gray matter tissues. These results indicate that potential alternative splicing events in CD4 T-cells may be highly important in MS and targeted for the development of specific treatment strategies.

The importance of alternative splicing events in MS has been highlighted within the last decade by several different approaches [9,10,77,78]. Our findings supported the previous evidence for the IL7R splice variant in MS as well as the dysregulation of RNA binding proteins and the involvement of the alternative types of myelin proteins in MS pathology [78]. In this study, we covered the shortfall of previous bulk RNA-seq analyses and shed light on commonly overlooked alternative splicing events in the transcriptome. While we showed that some of the known genes associated with MS and neurodegeneration were alternatively spliced, our analysis revealed many novel genes and alternative splicing events that suggest further research to better understand MS pathology, variable patient responses, and discover effective treatment strategies.

While the utilization of publicly available data is cost-effective, allows cross-cohort comparisons, and encourages open science culture, this approach also brought some limitations to our study. Sample size disparities across comparisons and the lack of information about patient diagnoses limited our ability to generalize our findings. We also identified ribosomal subunit protein alterations which are shown to be relevant to many diseases including MS; specifically, RPS6 was proposed as a potential biomarker [79]. However, the datasets from various research centers and countries may have used different methods for ribosomal depletion for the RNA-seq library preparation. Therefore, we would hesitate to make any comments on the ribosomal subunits. Additionally, due to the lack of enough samples for the comparison of CD8+ T-cells separately, we could only include those samples together with CD4+ T-cells. Finally, while rMATS is a robust method for alternative splicing identification from short read data, the full protein isoform identification would require long-read sequencing methods, such as iso-seq and deep-proteome sequencing [80,81].

## 4. Materials and Methods

### 4.1. Data Access

GEO [13] was searched for public bulk RNAseq data from postmortem MS patient brains. Eleven datasets were identified for analysis (Table 1). Raw fastq files were downloaded using “fastqdump” from the SRA Toolkit (version 3.0.0) (NCBI, Bethesda, Maryland, SRA-Tools https://github.com/ncbi/sra-tools (accessed on 30 May 2025)).

### 4.2. Differential Expression Analysis

Raw reads from each study were mapped to the human genome (hg38.p12) using the Spliced Transcripts Alignment to a Reference (STAR) aligner (version 2.6) [82]. Raw gene counts were determined using HTSeq-count (version 0.10.0) [83] and annotated with Ensembl (hg38.p12). Raw counts were normalized using the relative log expression method and filtered to exclude genes with fewer than 10 counts across all samples. Differential expression analysis was performed with DESeq2 [84] using a negative binomial regression model to analyze pairwise comparisons. Statistical significance was determined using a Benjamini–Hochberg adjusted false discovery rate (FDR) cutoff of 0.05.

### 4.3. Alternative Splicing Analysis

Replicate multivariate analysis of transcript splicing (rMATS version 3.2.5) [85] was used to identify differentially spliced genes. rMATS employs a modified generalized linear mixed model to identify differential splicing from RNA-seq data with replicates. Using both splice junction and exon body read counts as input, rMATS computes the percent-splicing index (PSI) and the FDR for five major types of splicing events: skipped exons (SEs), mutually exclusive exons (MXEs), retained introns (RIs), and 5′ and 3′ alternative splice sites (A5SS and A3SS).

### 4.4. Pearson Correlation Analysis

We computed Pearson correlation coefficients to assess the linear association between *p*-values of DEGs and ASEs using the cor() function in R (method = “pearson”) [86] 2012).

### 4.5. NEASE (Network Enrichment Method for Alternative Splicing Events) and Domain Interaction Graph Guided ExploreR (DIGGER)

We utilized NEASE (version 1.3.1) which detects the protein features affected by alternative splicing to identify the protein domains that were affected by alternative splicing events [34]. Then, we utilized DIGGER (version 2.0), which integrates protein–protein interactions and domain–domain interactions into a joint graph [87].

## Figures and Tables

**Table 1:** Eleven studies with bulk RNA-seq data from postmortem brain samples from MS patients.

Study ID	Summary	Study Site	Reference
GSE111972	Microglia from occipital cortex from non-MS controls (GM, *n* = 5) and MS patients GML (*n* = 5). Microglia from corpus callosum from non-MS controls (WM, *n* = 11) and MS patients (WML, *n* = 11)	Germany	[14]
GSE123496	Corpus callosum (WM *n* = 5, WML *n* = 5), frontal cortex (*n* = 5, *n* = 5), parietal cortex (*n* = 5, *n* = 5), hippocampus (*n* = 5, *n* = 5), and internal capsule (WM *n* = 5, WML *n* = 5) from non-MS control and MS patients	USA	[15]
I confiGSE137619	Choroid plexus from non-MS control (n = 6) and MS patients (*n* = 6)	Netherlands	[16]
GSE138614	WM (*n* = 25) from non-MS controls, NAWM (*n* = 21), and WML (*n* = 52) from MS patients	Denmark	[17]
GSE149326	NAGM (*n* = 11), GML (*n* = 11), NAWM (*n* = 11), WML (*n* = 10) from MS patients	Netherlands	[18]
GSE179427	NAWM (*n* = 31) and WML (*n* = 11) from MS patients, WM (*n* = 26) from non-MS controls	Netherlands	[19]
GSE207680	GM Cortex from non-MS controls (*n* = 3) and from PMS patients (*n* = 3)	Canada	[20]
GSE214334	NAWM from non-MS control (*n* = 7), RRMS (*n* = 3), SPMS (*n* = 4), and PPMS (*n* = 4) patients	Australia	[21]
GSE216028	NAGM (*n* = 11), GML (*n* = 5), NAWM (*n* = 14), WML (*n* = 10) CD4/CD8 T-cells from MS patients	Netherlands	[22]
GSE224377	NAWM and MS from MS patients (*n* = 9)	Belgium	[23]
GSE234700	Microglia from NAWM and WML from MS patients (*n* = 7)	Netherlands	[24]

White matter (WM), gray matter (GM), normal appearing white matter (NAWM), normal appearing gray matter (NAGM), white matter lesion (WML), gray matter lesion (GML), progressive MS (PMS), primary progressive MS (PPMS), secondary progressive MS (SPMS), and relapsing–remitting MS (RRMS).

**Table 2:** Twenty-five comparisons (numbered from C1 to C25) from eleven studies according to the diagnosis, brain region, tissue type, and cell type information for the available data.

Study ID	Experimental Group Diagnosis	Control Group Diagnosis	Brain Region	Tissue Type	Cell Type	Comparison Number
GSE111972	MS	Non-MS	Corpus Callosum	White Matter	Microglia	C1
GSE111972	MS	Non-MS	Occipital Cortex	Gray Matter	Microglia	C2
GSE123496	MS	Non-MS	Corpus Callosum	White Matter	Bulk	C3
GSE123496	MS	Non-MS	Internal Capsule	White Matter	Bulk	C4
GSE123496	MS	Non-MS	Frontal Cortex	Other	Bulk	C5
GSE123496	MS	Non-MS	Parietal Cortex	Other	Bulk	C6
GSE123496	MS	Non-MS	Hippocampus	Other	Bulk	C7
GSE137619	MS	Non-MS	Choroid Plexus	Other	Bulk	C8
GSE138614	MS	Non-MS	Not Specified	White Matter (AL)	Bulk	C9
GSE138614	MS	Non-MS	Not Specified	White Matter (RL)	Bulk	C10
GSE138614	MS	Non-MS	Not Specified	White Matter (IL)	Bulk	C11
GSE138614	MS	Non-MS	Not Specified	White Matter (CA)	Bulk	C12
GSE138614	MS	Non-MS	Not Specified	White Matter (NAWM)	Bulk	C13
GSE149326	MS	MS *	Not specified	White Matter	Bulk	C14
GSE149326	MS	MS *	Not specified	Gray Matter	Bulk	C15
GSE179427	MS	Non-MS	Not specified	White Matter (WML)	Bulk	C16
GSE179427	MS	Non-MS	Not specified	White Matter (NAWM)	Bulk	C17
GSE207680	PMS	Non-MS	Cortex	Gray Matter	Bulk	C18
GSE214334	PPMS	Non-MS	Not Specified	White Matter (NAWM)	Bulk	C19
GSE214334	SPMS	Non-MS	Not Specified	White Matter (NAWM)	Bulk	C20
GSE214334	RRMS	Non-MS	Not Specified	White Matter (NAWM)	Bulk	C21
GSE216028	MS	MS *	Not Specified	White Matter	T-cells (CD4+ and CD8+)	C22
GSE216028	MS	MS *	Not Specified	Gray Matter	T-cells (CD4+ and CD8+)	C23
GSE224377	MS	MS *	Not specified	White Matter	Bulk	C24
GSE234700	MS	MS *	Not Specified	White Matter	Microglia	C25

PMS: progressive MS, PPMS: primary progressive MS. SPMS: secondary progressive MS. RRMS: relapsing–remitting MS. AL: active lesion, RL: remyelinating lesion, IL: inactive lesion, CA: chronic active lesion. NAWM: normal appearing white matter. WML: white matter lesion. * The control groups for these studies were the normal appearing tissues from the same MS patients.

**Table 3:** The number of differentially expressed genes (DEGs) only, alternatively spliced genes (ASEs) only, and genes that are both DEG and ASE in each comparison.

Study ID	Comparison	DEG Only (Gene Count)	DEG and ASE (Gene Count—Percentage)	ASE Only (Gene Count)
GSE111972	C1	808	70—(4.46%)	691
GSE111972	C2	264	17—(1.59%)	691
GSE123496	C3	445	53—(3.32%)	1097
GSE123496	C4	444	26—(1.92%)	883
GSE123496	C5	10	0—(0%)	790
GSE123496	C6	4	0—(0%)	878
GSE123496	C7	47	0—(0%)	571
GSE137619	C8	11	0—(0%)	798
GSE138614	C9	6125	304—(4.27%)	687
GSE138614	C10	2907	263—(5.97%)	1233
GSE138614	C11	4937	401—(6.2%)	1126
GSE138614	C12	5731	234—(3.48%)	764
GSE138614	C13	719	32—(1.49%)	1403
GSE149326	C14	32	0—(0%)	4
GSE149326	C15	61	0—(0%)	4
GSE179427	C16	104	0—(0%)	64
GSE179427	C17	351	2—(0.48%)	65
GSE207680	C18	80	8—(0.72%)	1022
GSE214334	C19	1163	129—(4.19%)	1786
GSE214334	C20	4593	1355—(16.03%)	2504
GSE214334	C21	6	0—(0%)	912
GSE216028	C22	2	1—(0.32%)	458
GSE216028	C23	142	1—(0.29%)	200
GSE224377	C24	34	0—(0%)	3
GSE234700	C25	1053	0—(0%)	3

CC: corpus callosum. FC: frontal cortex. Hipp: hippocampus. PC: parietal cortex. IC: internal capsule. AL: active lesion. CA: chronic active lesion. IL: inactive lesion. RL: remyelinating lesion. NAWM: normal appearing white matter. WML: white matter lesion. CP: choroid plexus. PPMS: primary progressive MS. RRMS: relapsing–remitting MS. SPMS: secondary progressive MS.

**Table 4:** Studies and comparison numbers for diagnosis comparisons.

MS Lesions vs. Non-MS		Progressive MS vs. Non-MS		RRMS vs. Non-MS	
Study ID	Comparison Number	Study ID	Comparison Number	Study ID	Comparison Number
GSE111972	C1	GSE207680	C18	GSE214334	C21
GSE111972	C2	GSE214334	C19		
GSE123496	C3	GSE214334	C20		
GSE123496	C4				
GSE123496	C5				
GSE123496	C6				
GSE123496	C7				
GSE138614	C9				
GSE138614	C10				
GSE138614	C11				
GSE138614	C12				
GSE149326	C14				
GSE149326	C15				
GSE179427	C16				
GSE207680	C18				

**Table 5:** Studies and comparison numbers for brain region comparisons.

Corpus Callosum		IC, FC, PC, Hipp		Occipital Cortex		Choroid Plexus	
Study ID	Comparison Number	Study ID	Comparison Number (Respectively IC, FC, PC, Hipp)	Study ID	Comparison Number	Study ID	Comparison Number
GSE111972	C1	GSE123496	C4	GSE111972	C2	GSE137619	C8
GSE123496	C3	GSE123496	C5				
		GSE123496	C6				
		GSE123496	C7				

IC: internal capsule, FC: frontal cortex, PC: parietal cortex, Hipp: hippocampus.

**Table 6:** Genes that have identical alternative splicing events (ASEs) in CC from two studies (GSE123496 and GSE111972).

Corpus Callosum		
Gene Symbol	Gene Description	ASE
*A2M*	alpha-2-macroglobulin	RI
*ACSL1*	acyl-CoA synthetase long chain family member 1	MXE
*ADAM28*	ADAM metallopeptidase domain 28	SE
*AKAP8L*	A-kinase anchoring protein 8 like	RI
*AMPD3*	adenosine monophosphate deaminase 3	SE
*BIN1*	bridging integrator 1	SE
*CLK1*	CDC like kinase 1	SE, RI
*COX4I1*	cytochrome c oxidase subunit 4I1	RI
*DDX5*	DEAD-box helicase 5	SE
*DENND5A*	DENN domain containing 5A	SE
*DNAJB2*	DnaJ heat shock protein family (Hsp40) member B2	RI
*EEF1D*	eukaryotic translation elongation factor 1 delta	RI
*EPB41L2*	erythrocyte membrane protein band 4.1 like 2	SE
*FTH1*	ferritin heavy chain 1	RI
*HEXA*	hexosaminidase subunit alpha	SE
*HNRNPH1*	heterogeneous nuclear ribonucleoprotein H1	SE, RI
*HNRNPH3*	heterogeneous nuclear ribonucleoprotein H3	SE
*INTS6*	integrator complex subunit 6	SE
*LZTS2*	leucine zipper tumor suppressor 2	SE
*NDUFV3*	NADH:ubiquinone oxidoreductase subunit V3	SE
*PHB2*	prohibitin 2	SE
*QKI*	KH domain containing RNA binding	A3SS
*RGS2*	regulator of G protein signaling 2	SE
*RPL10A*	ribosomal protein L10a	RI
*RPL28*	ribosomal protein L28	RI
*RPS9*	ribosomal protein S9	A3SS
*SNHG1*	small nucleolar RNA host gene 1	RI
*SPP1*	secreted phosphoprotein 1	SE
*TMEM59*	transmembrane protein 59	SE
*TPM3*	tropomyosin 3	SE
*TPP1*	tripeptidyl peptidase 1	RI
*YBX3*	Y-box binding protein 3	RI
*YWHAB*	tyrosine 3-monooxygenase/tryptophan 5-monooxygenase activation protein beta	RI

Skipped exon (SE), mutually exclusive exons (MXEs), retained introns (RIs), and 5′ and 3′ alternative splice sites (A5SS and A3SS).

**Table 7:** Studies and comparison numbers for tissue type comparisons.

NAWM vs. WM		WML vs. WM		GML vs. GM	
Study ID	Comparison Number	Study ID	Comparison Number	Study ID	Comparison Number
GSE138614	C13	GSE111972	C1	GSE111972	C2
GSE179427	C17	GSE123496-CC	C3	GSE207680	C18
GSE214334-PP	C19	GSE123496-IC	C4		
GSE214334-SP	C20	GSE138614	C9–C12		
GSE214334-RR	C21				

NAWM: normal-appearing white matter, WM: white matter, WML: white matter lesion, GML: gray matter lesion, GM: gray matter.

**Table 8:** Studies and comparison numbers for cell type comparisons.

Microglia		T-Cells (CD4+/CD8+)	
Study ID	Comparison Number	Study ID	Comparison Number
GSE111972	C1	GSE216028	C22
GSE111972	C2	GSE216028	C23
GSE234700	C25		

**Table 9:** Genes that have identical alternative splicing events (ASEs) in CD4/CD8 T-cells in both white matter and gray matter lesions from the study GSE216028.

CD8/CD4 T-Cells		
Gene Symbol	Gene Description	ASE
*AL590764.2*	NA	SE
*ARGLU1*	arginine and glutamate rich 1	RI
*ARHGEF1*	Rho guanine nucleotide exchange factor 1	A3SS
*ARL6IP4*	ADP ribosylation factor like GTPase 6 interacting protein 4	RI
*ARPC2*	actin related protein 2/3 complex subunit 2	SE
*BIN2*	bridging integrator 2	SE
*C9orf78*	chromosome 9 open reading frame 78	SE
*CD37*	CD37 molecule	SE, A3SS
*CD96*	CD96 molecule	SE
*CD99*	CD99 molecule (Xg blood group)	SE, A3SS
*CDK5RAP3*	CDK5 regulatory subunit associated protein 3	RI
*CENPT*	centromere protein T	A3SS, RI
*CHURC1*	Churchill domain containing 1	SE
*CIRBP*	cold-inducible RNA binding protein	SE
*COX5B*	cytochrome c oxidase subunit 5B	SE
*CPNE1*	copine 1	RI
*DDX5*	DEAD-box helicase 5	RI
*DENND2D*	DENN domain containing 2D	SE
*EIF1*	eukaryotic translation initiation factor 1	SE
*ELOB*	elongin B	RI
*EMP3*	epithelial membrane protein 3	SE
*EXOSC8*	exosome component 8	A3SS
*GAS5*	growth arrest specific 5	RI
*GLIPR1*	GLI pathogenesis related 1	SE
*GMFG*	glia maturation factor gamma	SE
*GSTK1*	glutathione S-transferase kappa 1	SE
*GTF3A*	general transcription factor IIIA	SE
*GZMA*	granzyme A	SE
*H3-3B*	H3.3 histone B	RI
*HLA-A*	major histocompatibility complex, class I, A	RI
*HLA-B*	major histocompatibility complex, class I, B	RI
*HNRNPA1*	heterogeneous nuclear ribonucleoprotein A1	SE
*HNRNPC*	heterogeneous nuclear ribonucleoprotein C	A3SS
*HNRNPU*	heterogeneous nuclear ribonucleoprotein U	RI
*HSPB1*	heat shock protein family B (small) member 1	A5SS
*HSPE1*	heat shock protein family E (Hsp10) member 1	SE
*IL2RG*	interleukin 2 receptor subunit gamma	RI
*IL32*	interleukin 32	SE, A3SS
*IL7R*	interleukin 7 receptor	SE
*ILF3*	interleukin enhancer binding factor 3	SE
*ISCU*	iron–sulfur cluster assembly enzyme	SE
*LCK*	LCK proto-oncogene, Src family tyrosine kinase	A3SS
*LIMD2*	LIM domain containing 2	A3SS, RI
*MYL6*	myosin light chain 6	SE, A5SS, A3SS, RI
*NACA*	nascent polypeptide-associated complex subunit alpha	RI
*NDUFA11*	NADH:ubiquinone oxidoreductase subunit A11	SE, RI
*NDUFA3*	NADH:ubiquinone oxidoreductase subunit A3	SE
*OAZ1*	ornithine decarboxylase antizyme 1	RI
*PABPC1*	poly(A) binding protein cytoplasmic 1	A5SS
*PCED1B-AS1*	PCED1B antisense RNA 1	SE, A5SS
*PFDN5*	prefoldin subunit 5	RI
*PPIA*	peptidylprolyl isomerase A	SE
*PTPN6*	protein tyrosine phosphatase non-receptor type 6	A3SS
*RACK1*	receptor for activated C kinase 1	RI
*RBM39*	RNA binding motif protein 39	RI
*RPL10*	ribosomal protein L10	RI
*RPL10A*	ribosomal protein L10a	RI
*RPL13A*	ribosomal protein L13a	RI
*RPL28*	ribosomal protein L28	RI
*RPL3*	ribosomal protein L3	RI
*RPL31*	ribosomal protein L31	RI
*RPL4*	ribosomal protein L4	RI
*RPL41*	ribosomal protein L41	A5SS
*RPLP1*	ribosomal protein lateral stalk subunit P1	SE
*RPS11*	ribosomal protein S11	RI
*RPS12*	ribosomal protein S12	A5SS
*RPS15*	ribosomal protein S15	A3SS
*RPS2*	ribosomal protein S2	RI
*RPS20*	ribosomal protein S20	RI
*RPS28*	ribosomal protein S28	RI
*RPS3*	ribosomal protein S3	SE, A5SS
*RPS9*	ribosomal protein S9	SE
*SKAP1*	src kinase associated phosphoprotein 1	SE
*SNRPN*	small nuclear ribonucleoprotein polypeptide N	SE, A3SS
*SPSB3*	splA/ryanodine receptor domain and SOCS box containing 3	RI
*SRRM1*	serine and arginine repetitive matrix 1	RI
*SYF2*	SYF2 pre-mRNA splicing factor	SE
*TPM3*	tropomyosin 3	SE
*TPT1*	tumor protein, translationally-controlled 1	SE
*UQCRB*	ubiquinol-cytochrome c reductase binding protein	SE
*VPS29*	VPS29 retromer complex component	SE

Skipped exon (SE), mutually exclusive exons (MXEs), retained introns (RI), and 5′ and 3′ alternative splice sites (A5SS and A3SS).

## Data Availability

The samples utilized in this analysis are publicly available in GEO. The differential expression and differential splicing files are provided at https://doi.org/10.6084/m9.figshare.29395844.v1.

## Supplementary Materials

The following supporting information can be downloaded at https://www.mdpi.com/article/10.3390/ijms26178195/s1.
