# Supplementary material for: Systematic Analysis of Alternative Splicing in Transcriptomes of Multiple Sclerosis Patient Brain Samples
Source: Int J Mol Sci. 2025 Aug 23;26(17):8195. doi: 10.3390/ijms26178195 (PMC12428622; doi:10.3390/ijms26178195)
Supplement: Supplementary file 1 [file ijms-26-08195-s001.zip › Figure S1.pdf]

Figure S1

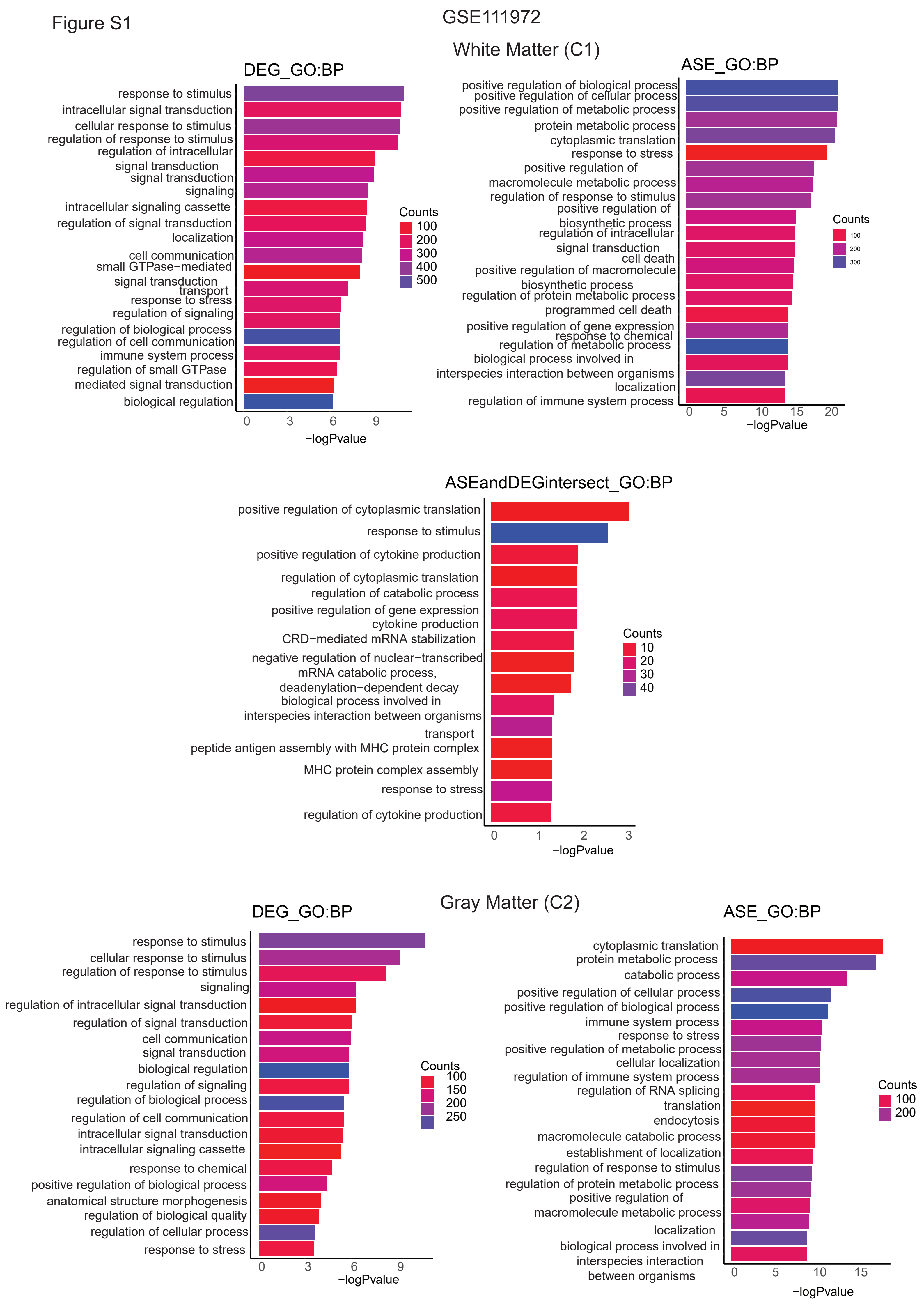
GSE111972
White Matter (C1)
DEG_GO:BP
response to stimulus
intracellular signal transduction
cellular response to stimulus
regulation of response to stimulus
regulation of intracellular signal transduction
signal transduction
signaling
intracellular signaling cassette
regulation of signal transduction
localization
cell communication
small GTPase−mediated signal transduction
transport
response to stress
regulation of signaling
regulation of biological process
regulation of cell communication
immune system process
regulation of small GTPase mediated signal transduction
biological regulation
0 3 6 9
−logPvalue
Counts
100
200
300
400
500
ASE_GO:BP
positive regulation of biological process
positive regulation of cellular process
positive regulation of metabolic process
protein metabolic process
cytoplasmic translation
response to stress
positive regulation of macromolecule metabolic process
regulation of response to stimulus
positive regulation of biosynthetic process
regulation of intracellular signal transduction
cell death
positive regulation of macromolecule biosynthetic process
regulation of protein metabolic process
programmed cell death
positive regulation of gene expression
response to chemical
regulation of metabolic process
biological process involved in interspecies interaction between organisms
localization
regulation of immune system process
0 5 10 15 20
−logPvalue
Counts
100
200
300
ASEandDEGintersect_GO:BP
positive regulation of cytoplasmic translation
response to stimulus
positive regulation of cytokine production
regulation of cytoplasmic translation
regulation of catabolic process
positive regulation of gene expression
cytokine production
CRD−mediated mRNA stabilization
negative regulation of nuclear−transcribed mRNA catabolic process, deadenylation−dependent decay
biological process involved in interspecies interaction between organisms
transport
peptide antigen assembly with MHC protein complex
MHC protein complex assembly
response to stress
regulation of cytokine production
0 1 2 3
−logPvalue
Counts
10
20
30
40
Gray Matter (C2)
DEG_GO:BP
response to stimulus
cellular response to stimulus
regulation of response to stimulus
signaling
regulation of intracellular signal transduction
regulation of signal transduction
cell communication
signal transduction
biological regulation
regulation of signaling
regulation of biological process
regulation of cell communication
intracellular signal transduction
intracellular signaling cassette
response to chemical
positive regulation of biological process
anatomical structure morphogenesis
regulation of biological quality
regulation of cellular process
response to stress
0 3 6 9
−logPvalue
Counts
100
150
200
250
ASE_GO:BP
cytoplasmic translation
protein metabolic process
catabolic process
positive regulation of cellular process
positive regulation of biological process
immune system process
response to stress
positive regulation of metabolic process
cellular localization
regulation of immune system process
regulation of RNA splicing
translation
endocytosis
macromolecule catabolic process
establishment of localization
regulation of response to stimulus
regulation of protein metabolic process
positive regulation of macromolecule metabolic process
localization
biological process involved in interspecies interaction between organisms
0 5 10 15
−logPvalue
Counts
100
200

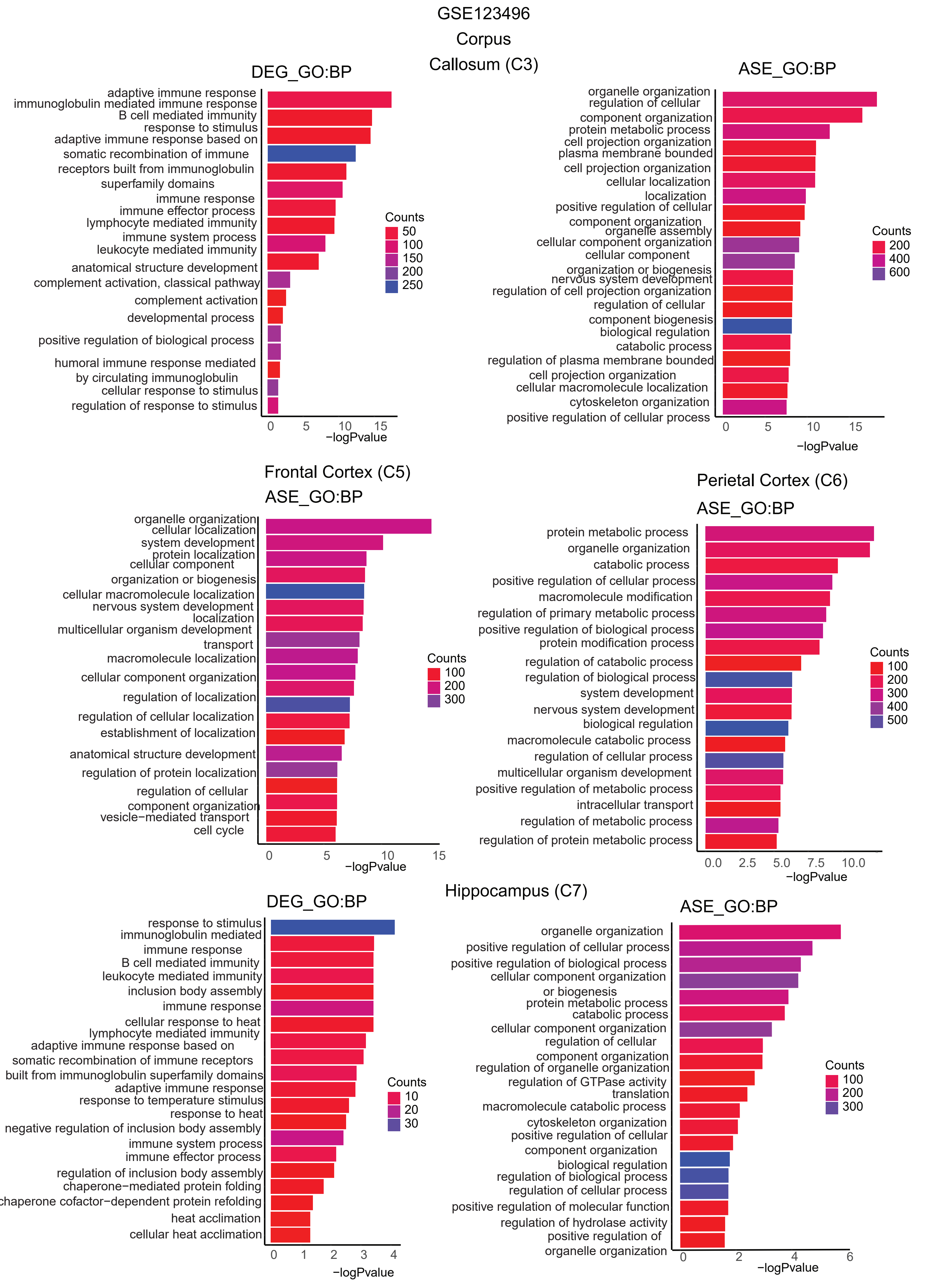

GSE123496
Corpus Callosum (C3)
DEG_GO:BP
adaptive immune response
immunoglobulin mediated immune response
B cell mediated immunity
response to stimulus
adaptive immune response based on somatic recombination of immune receptors built from immunoglobulin superfamily domains
immune response
immune effector process
lymphocyte mediated immunity
immune system process
leukocyte mediated immunity
anatomical structure development
complement activation, classical pathway
complement activation
developmental process
positive regulation of biological process
humoral immune response mediated by circulating immunoglobulin
cellular response to stimulus
regulation of response to stimulus
Counts
50
100
150
200
250
−logPvalue
ASE_GO:BP
organelle organization
regulation of cellular component organization
protein metabolic process
cell projection organization
plasma membrane bounded cell projection organization
cellular localization
localization
positive regulation of cellular component organization
organelle assembly
cellular component organization
cellular component organization or biogenesis
nervous system development
regulation of cell projection organization
regulation of cellular component biogenesis
biological regulation
catabolic process
regulation of plasma membrane bounded cell projection organization
cellular macromolecule localization
cytoskeleton organization
positive regulation of cellular process
Counts
200
400
600
−logPvalue
Frontal Cortex (C5)
ASE_GO:BP
organelle organization
cellular localization
system development
protein localization
cellular component organization or biogenesis
cellular macromolecule localization
nervous system development
localization
multicellular organism development
transport
macromolecule localization
cellular component organization
regulation of localization
regulation of cellular localization
establishment of localization
anatomical structure development
regulation of protein localization
regulation of cellular component organization
vesicle−mediated transport
cell cycle
Counts
100
200
300
−logPvalue
Perietal Cortex (C6)
ASE_GO:BP
protein metabolic process
organelle organization
catabolic process
positive regulation of cellular process
macromolecule modification
regulation of primary metabolic process
positive regulation of biological process
protein modification process
regulation of catabolic process
regulation of biological process
system development
nervous system development
biological regulation
macromolecule catabolic process
regulation of cellular process
multicellular organism development
positive regulation of metabolic process
intracellular transport
regulation of metabolic process
regulation of protein metabolic process
Counts
100
200
300
400
500
−logPvalue
Hippocampus (C7)
DEG_GO:BP
response to stimulus
immunoglobulin mediated immune response
B cell mediated immunity
leukocyte mediated immunity
inclusion body assembly
immune response
cellular response to heat
lymphocyte mediated immunity
adaptive immune response based on somatic recombination of immune receptors built from immunoglobulin superfamily domains
adaptive immune response
response to temperature stimulus
response to heat
negative regulation of inclusion body assembly
immune system process
immune effector process
regulation of inclusion body assembly
chaperone−mediated protein folding
chaperone cofactor−dependent protein refolding
heat acclimation
cellular heat acclimation
Counts
10
20
30
−logPvalue
ASE_GO:BP
organelle organization
positive regulation of cellular process
positive regulation of biological process
cellular component organization or biogenesis
protein metabolic process
catabolic process
cellular component organization
regulation of cellular component organization
regulation of organelle organization
regulation of GTPase activity
translation
macromolecule catabolic process
cytoskeleton organization
positive regulation of cellular component organization
biological regulation
regulation of biological process
regulation of cellular process
positive regulation of molecular function
regulation of hydrolase activity
positive regulation of organelle organization
Counts
100
200
300
−logPvalue

GSE123496 cont'd

Internal Capsule (C4)

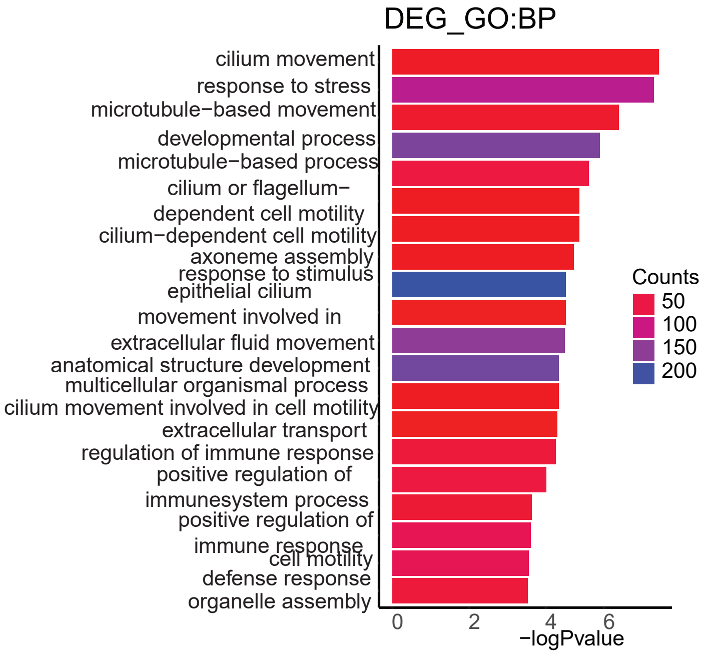

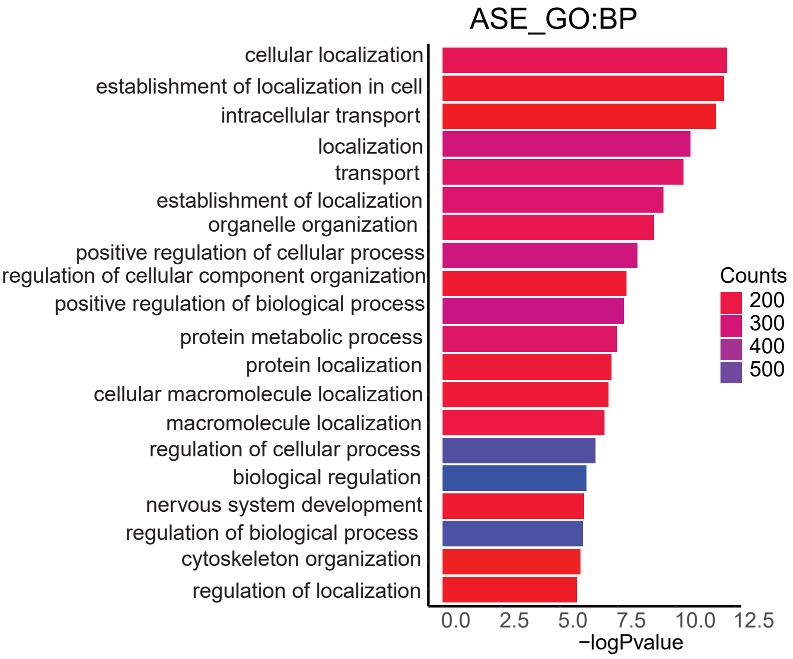

GSE138614

AL vs WM (C9)

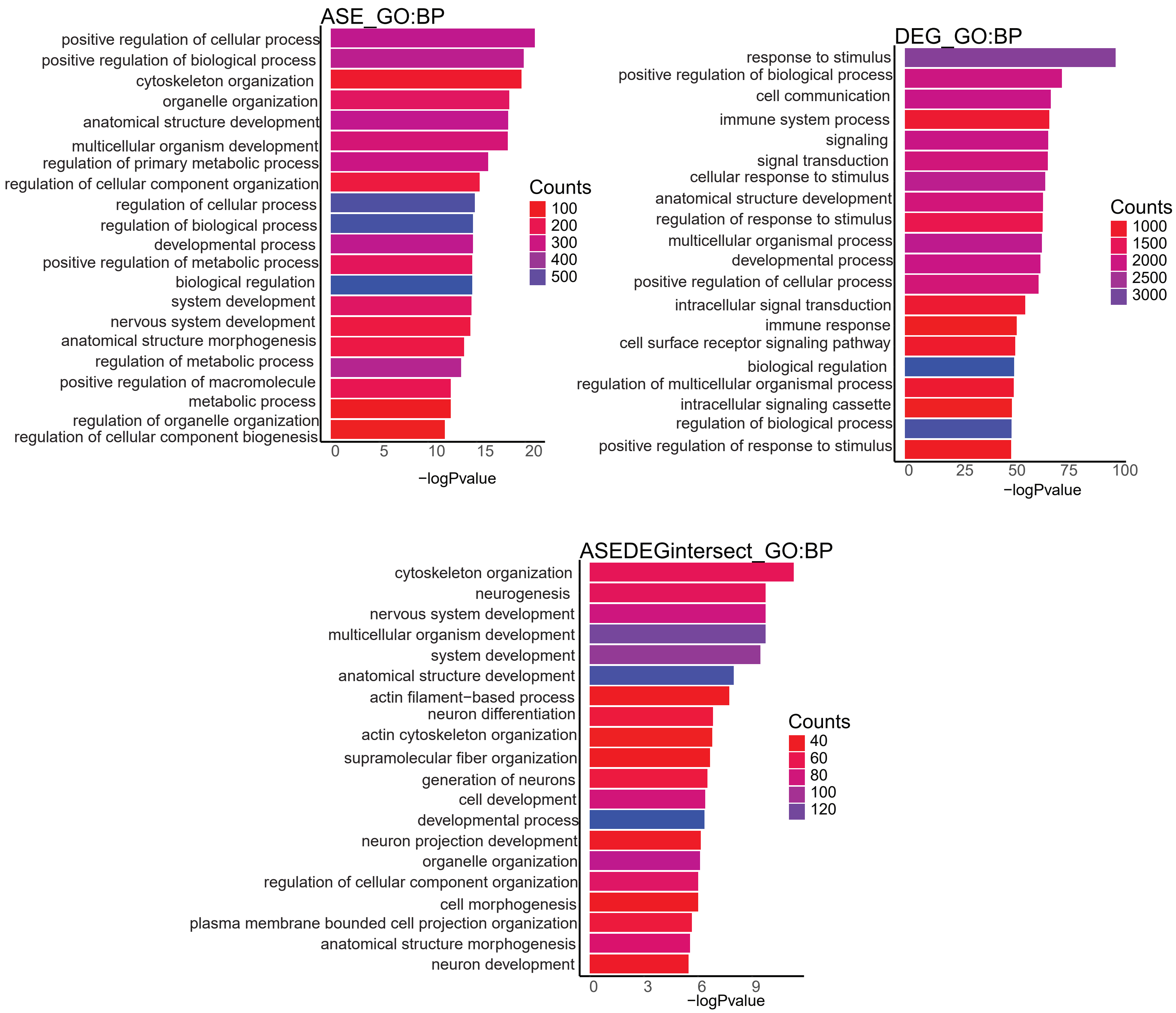

GSE138614

IL vs WM (C11)

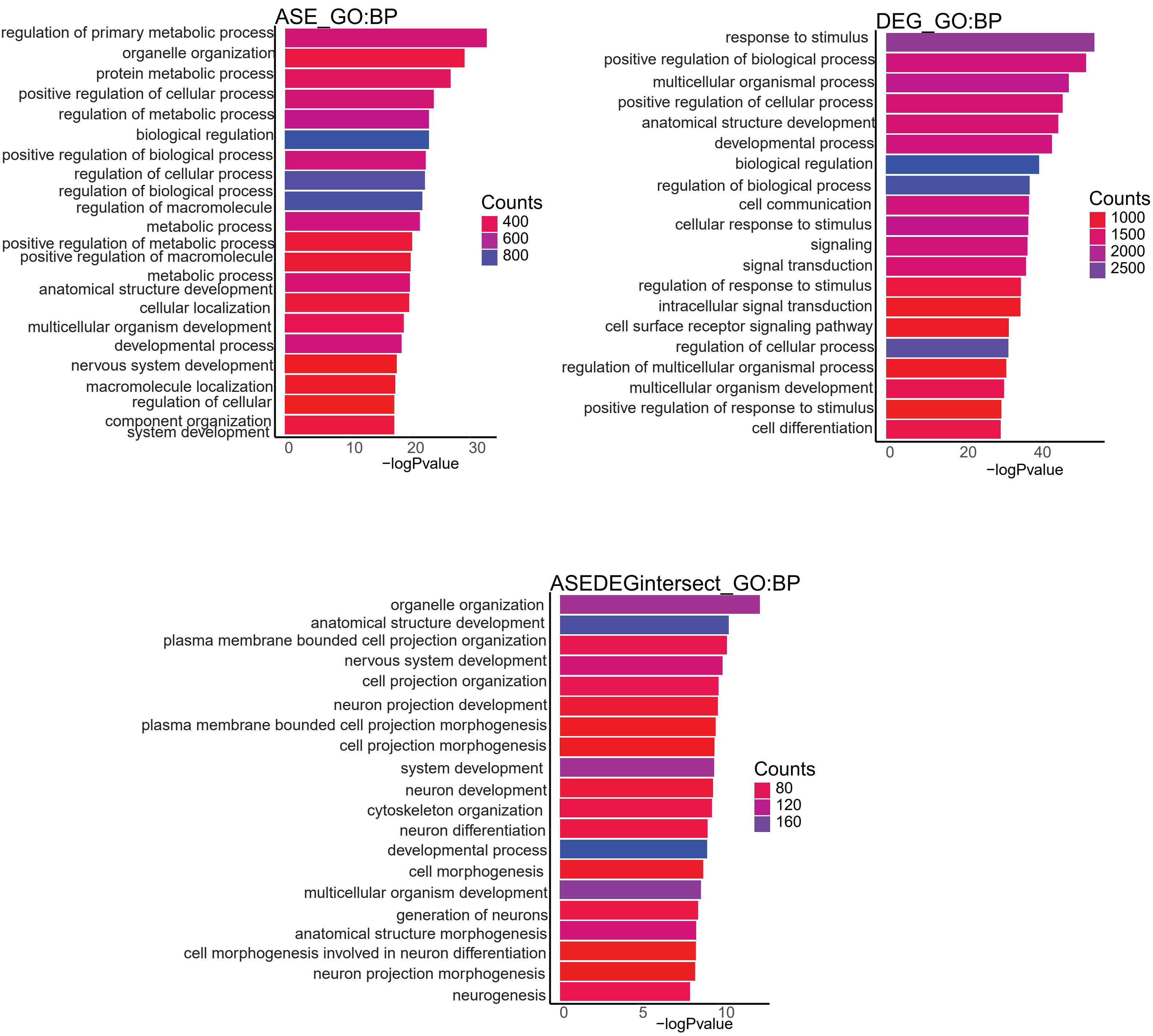

GSE138614

CA vs WM (C12)

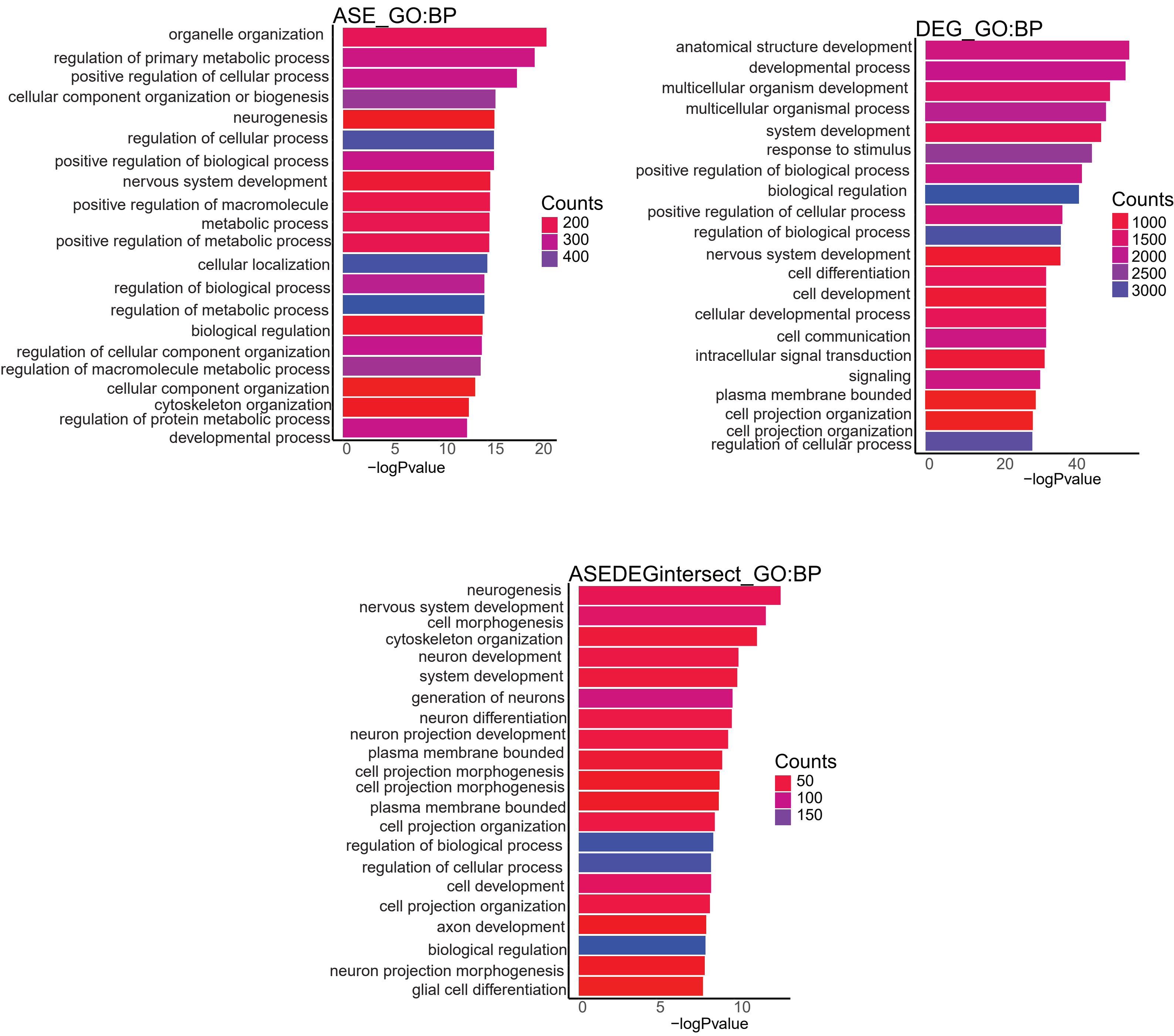

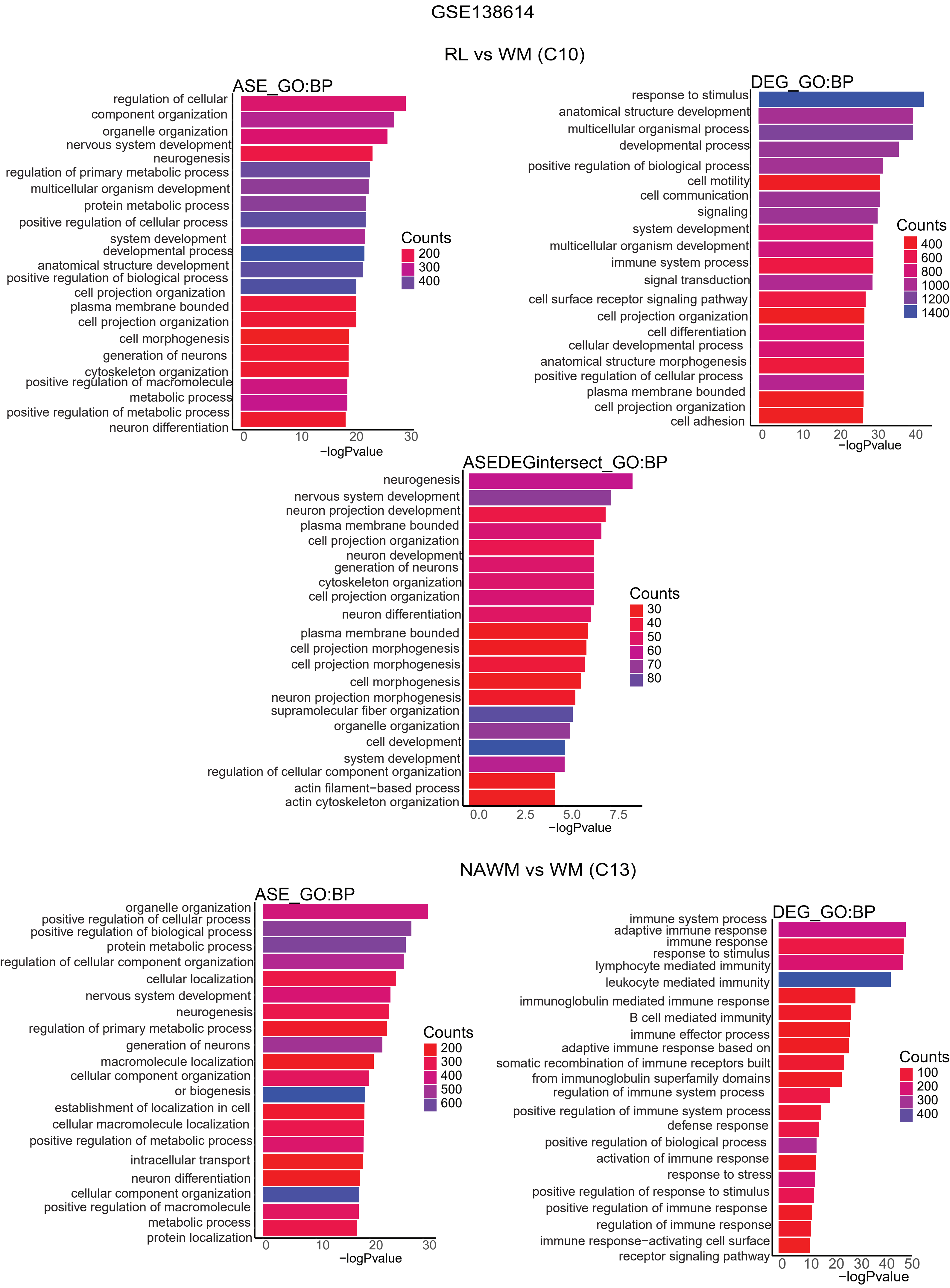

GSE138614
RL vs WM (C10)
ASE_GO:BP
regulation of cellular
component organization
organelle organization
nervous system development
neurogenesis
regulation of primary metabolic process
multicellular organism development
protein metabolic process
positive regulation of cellular process
system development
developmental process
anatomical structure development
positive regulation of biological process
cell projection organization
plasma membrane bounded
cell projection organization
cell morphogenesis
generation of neurons
cytoskeleton organization
positive regulation of macromolecule
metabolic process
positive regulation of metabolic process
neuron differentiation
0 10 20 30
−logPvalue
Counts
200
300
400
DEG_GO:BP
response to stimulus
anatomical structure development
multicellular organismal process
developmental process
positive regulation of biological process
cell motility
cell communication
signaling
system development
multicellular organism development
immune system process
signal transduction
cell surface receptor signaling pathway
cell projection organization
cell differentiation
cellular developmental process
anatomical structure morphogenesis
positive regulation of cellular process
plasma membrane bounded
cell projection organization
cell adhesion
0 10 20 30 40
−logPvalue
Counts
400
600
800
1000
1200
1400
ASEDEGintersect_GO:BP
neurogenesis
nervous system development
neuron projection development
plasma membrane bounded
cell projection organization
neuron development
generation of neurons
cytoskeleton organization
cell projection organization
neuron differentiation
plasma membrane bounded
cell projection morphogenesis
cell projection morphogenesis
cell morphogenesis
neuron projection morphogenesis
supramolecular fiber organization
organelle organization
cell development
system development
regulation of cellular component organization
actin filament−based process
actin cytoskeleton organization
0.0 2.5 5.0 7.5
−logPvalue
Counts
30
40
50
60
70
80
NAWM vs WM (C13)
ASE_GO:BP
organelle organization
positive regulation of cellular process
positive regulation of biological process
protein metabolic process
regulation of cellular component organization
cellular localization
nervous system development
neurogenesis
regulation of primary metabolic process
generation of neurons
macromolecule localization
cellular component organization
or biogenesis
establishment of localization in cell
cellular macromolecule localization
positive regulation of metabolic process
intracellular transport
neuron differentiation
cellular component organization
positive regulation of macromolecule
metabolic process
protein localization
0 10 20 30
−logPvalue
Counts
200
300
400
500
600
DEG_GO:BP
immune system process
adaptive immune response
immune response
response to stimulus
lymphocyte mediated immunity
leukocyte mediated immunity
immunoglobulin mediated immune response
B cell mediated immunity
immune effector process
adaptive immune response based on
somatic recombination of immune receptors built
from immunoglobulin superfamily domains
regulation of immune system process
positive regulation of immune system process
defense response
positive regulation of biological process
activation of immune response
response to stress
positive regulation of response to stimulus
positive regulation of immune response
regulation of immune response
immune response−activating cell surface
receptor signaling pathway
0 10 20 30 40 50
−logPvalue
Counts
100
200
300
400

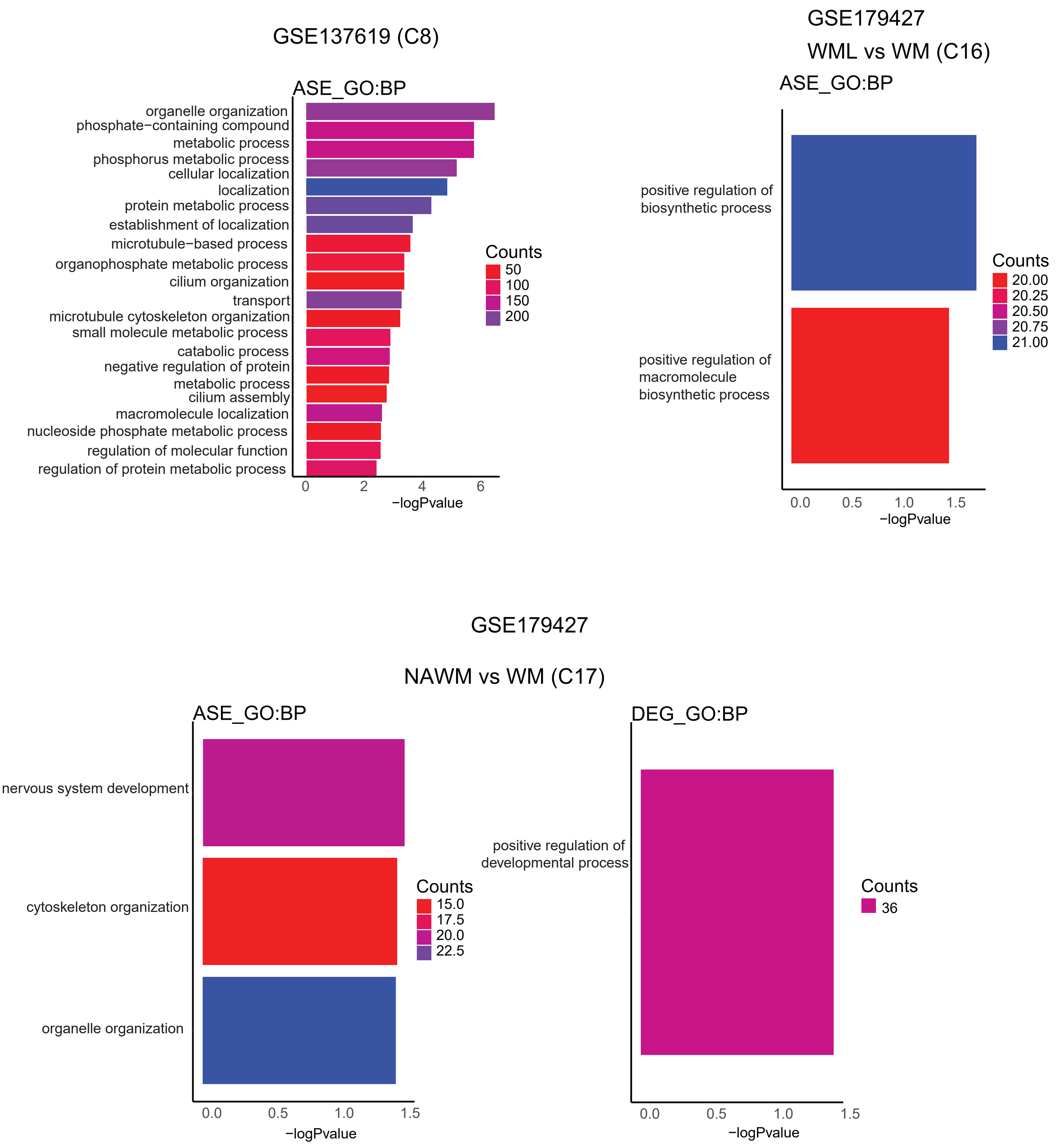
GSE137619 (C8)
ASE_GO:BP
organelle organization
phosphate−containing compound metabolic process
phosphorus metabolic process
cellular localization
localization
protein metabolic process
establishment of localization
microtubule−based process
organophosphate metabolic process
cilium organization
transport
microtubule cytoskeleton organization
small molecule metabolic process
catabolic process
negative regulation of protein metabolic process
cilium assembly
macromolecule localization
nucleoside phosphate metabolic process
regulation of molecular function
regulation of protein metabolic process
0
2
4
6
−logPvalue
Counts
50
100
150
200
GSE179427
WML vs WM (C16)
ASE_GO:BP
positive regulation of biosynthetic process
positive regulation of macromolecule biosynthetic process
0.0
0.5
1.0
1.5
−logPvalue
Counts
20.00
20.25
20.50
20.75
21.00
GSE179427
NAWM vs WM (C17)
ASE_GO:BP
nervous system development
cytoskeleton organization
organelle organization
0.0
0.5
1.0
1.5
−logPvalue
Counts
15.0
17.5
20.0
22.5
DEG_GO:BP
positive regulation of developmental process
0.0
0.5
1.0
1.5
−logPvalue
Counts
36

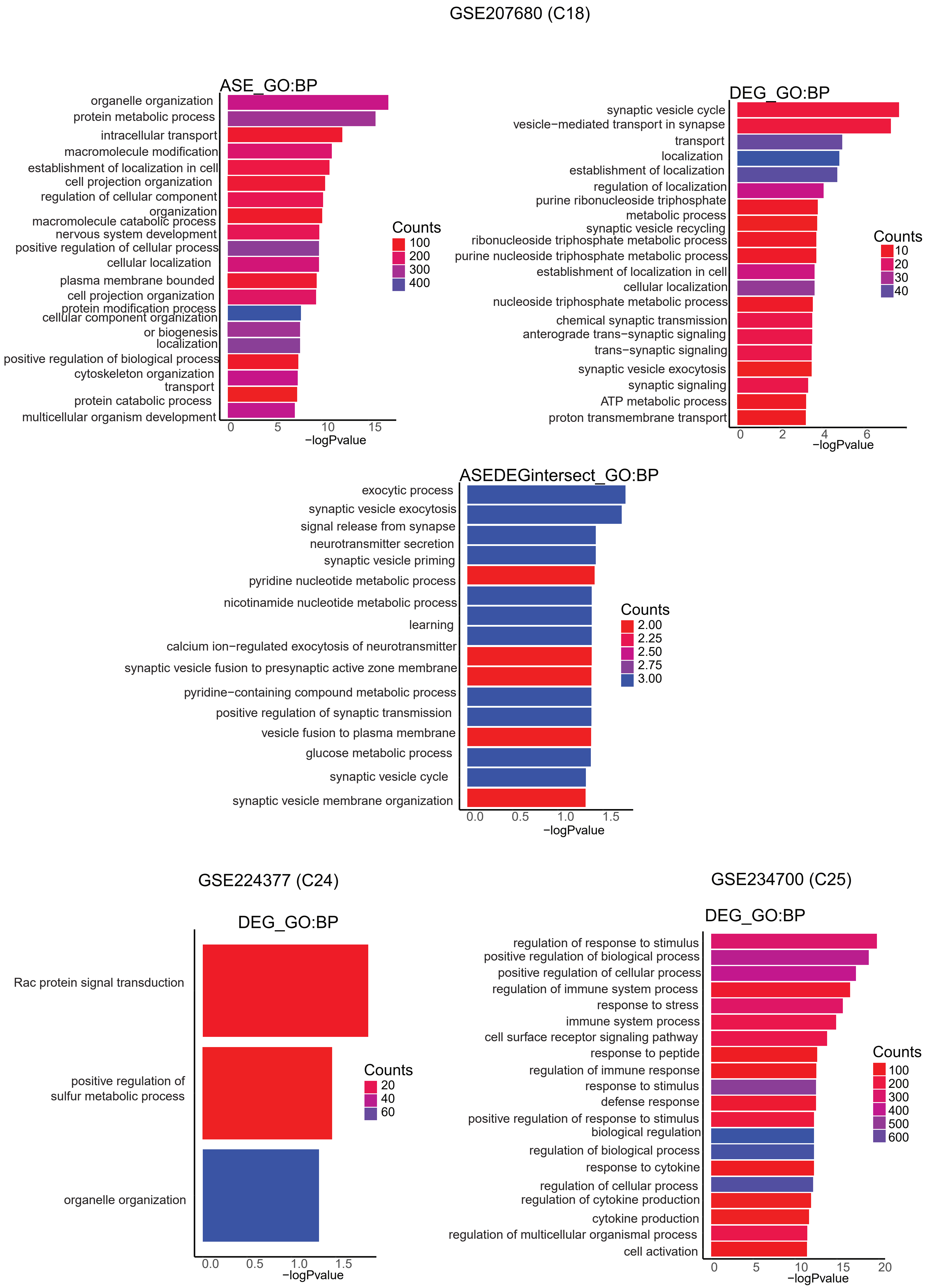
GSE207680 (C18)
ASE_GO:BP
organelle organization
protein metabolic process
intracellular transport
macromolecule modification
establishment of localization in cell
cell projection organization
regulation of cellular component organization
macromolecule catabolic process
nervous system development
positive regulation of cellular process
cellular localization
plasma membrane bounded cell projection organization
protein modification process
cellular component organization or biogenesis
localization
positive regulation of biological process
cytoskeleton organization
transport
protein catabolic process
multicellular organism development
0
5
10
15
−logPvalue
Counts
100
200
300
400
DEG_GO:BP
synaptic vesicle cycle
vesicle−mediated transport in synapse
transport
localization
establishment of localization
regulation of localization
purine ribonucleoside triphosphate metabolic process
synaptic vesicle recycling
ribonucleoside triphosphate metabolic process
purine nucleoside triphosphate metabolic process
establishment of localization in cell
cellular localization
nucleoside triphosphate metabolic process
chemical synaptic transmission
anterograde trans−synaptic signaling
trans−synaptic signaling
synaptic vesicle exocytosis
synaptic signaling
ATP metabolic process
proton transmembrane transport
0
2
4
6
−logPvalue
Counts
10
20
30
40
ASEDEGintersect_GO:BP
exocytic process
synaptic vesicle exocytosis
signal release from synapse
neurotransmitter secretion
synaptic vesicle priming
pyridine nucleotide metabolic process
nicotinamide nucleotide metabolic process
learning
calcium ion−regulated exocytosis of neurotransmitter
synaptic vesicle fusion to presynaptic active zone membrane
pyridine−containing compound metabolic process
positive regulation of synaptic transmission
vesicle fusion to plasma membrane
glucose metabolic process
synaptic vesicle cycle
synaptic vesicle membrane organization
0.0
0.5
1.0
1.5
−logPvalue
Counts
2.00
2.25
2.50
2.75
3.00
GSE224377 (C24)
DEG_GO:BP
Rac protein signal transduction
positive regulation of sulfur metabolic process
organelle organization
0.0
0.5
1.0
1.5
−logPvalue
Counts
20
40
60
GSE234700 (C25)
DEG_GO:BP
regulation of response to stimulus
positive regulation of biological process
positive regulation of cellular process
regulation of immune system process
response to stress
immune system process
cell surface receptor signaling pathway
response to peptide
regulation of immune response
response to stimulus
defense response
positive regulation of response to stimulus
biological regulation
regulation of biological process
response to cytokine
regulation of cellular process
regulation of cytokine production
cytokine production
regulation of multicellular organismal process
cell activation
0
5
10
15
20
−logPvalue
Counts
100
200
300
400
500
600

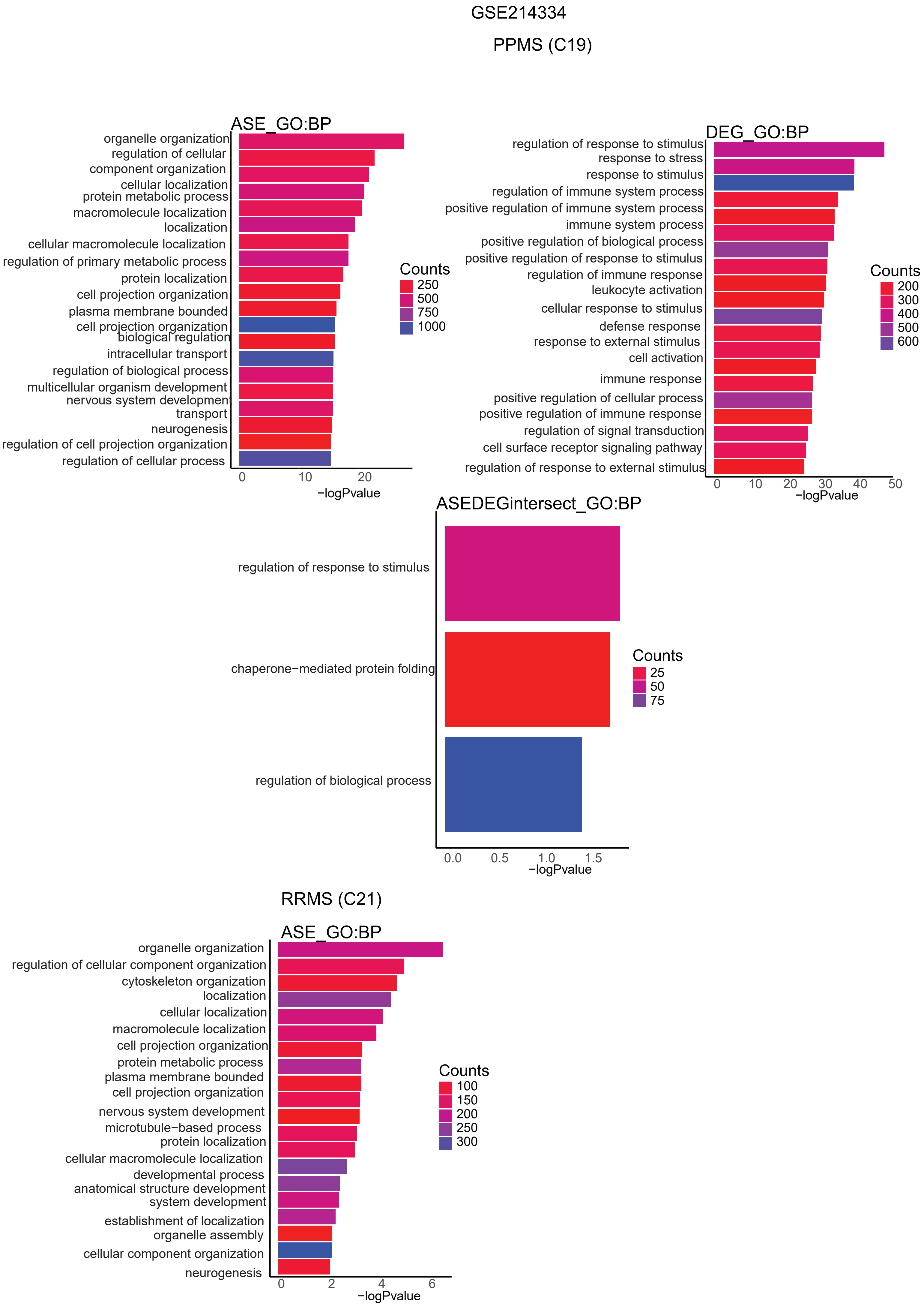
GSE214334
PPMS (C19)
ASE_GO:BP
organelle organization
regulation of cellular
component organization
cellular localization
protein metabolic process
macromolecule localization
localization
cellular macromolecule localization
regulation of primary metabolic process
protein localization
cell projection organization
plasma membrane bounded
cell projection organization
biological regulation
intracellular transport
regulation of biological process
multicellular organism development
nervous system development
transport
neurogenesis
regulation of cell projection organization
regulation of cellular process
0
10
20
−logPvalue
Counts
250
500
750
1000
DEG_GO:BP
regulation of response to stimulus
response to stress
response to stimulus
regulation of immune system process
positive regulation of immune system process
immune system process
positive regulation of biological process
positive regulation of response to stimulus
regulation of immune response
leukocyte activation
cellular response to stimulus
defense response
response to external stimulus
cell activation
immune response
positive regulation of cellular process
positive regulation of immune response
regulation of signal transduction
cell surface receptor signaling pathway
regulation of response to external stimulus
0
10
20
30
40
50
−logPvalue
Counts
200
300
400
500
600
ASEDEGintersect_GO:BP
regulation of response to stimulus
chaperone−mediated protein folding
regulation of biological process
0.0
0.5
1.0
1.5
−logPvalue
Counts
25
50
75
RRMS (C21)
ASE_GO:BP
organelle organization
regulation of cellular component organization
cytoskeleton organization
localization
cellular localization
macromolecule localization
cell projection organization
protein metabolic process
plasma membrane bounded
cell projection organization
nervous system development
microtubule−based process
protein localization
cellular macromolecule localization
developmental process
anatomical structure development
system development
establishment of localization
organelle assembly
cellular component organization
neurogenesis
0
2
4
6
−logPvalue
Counts
100
150
200
250
300

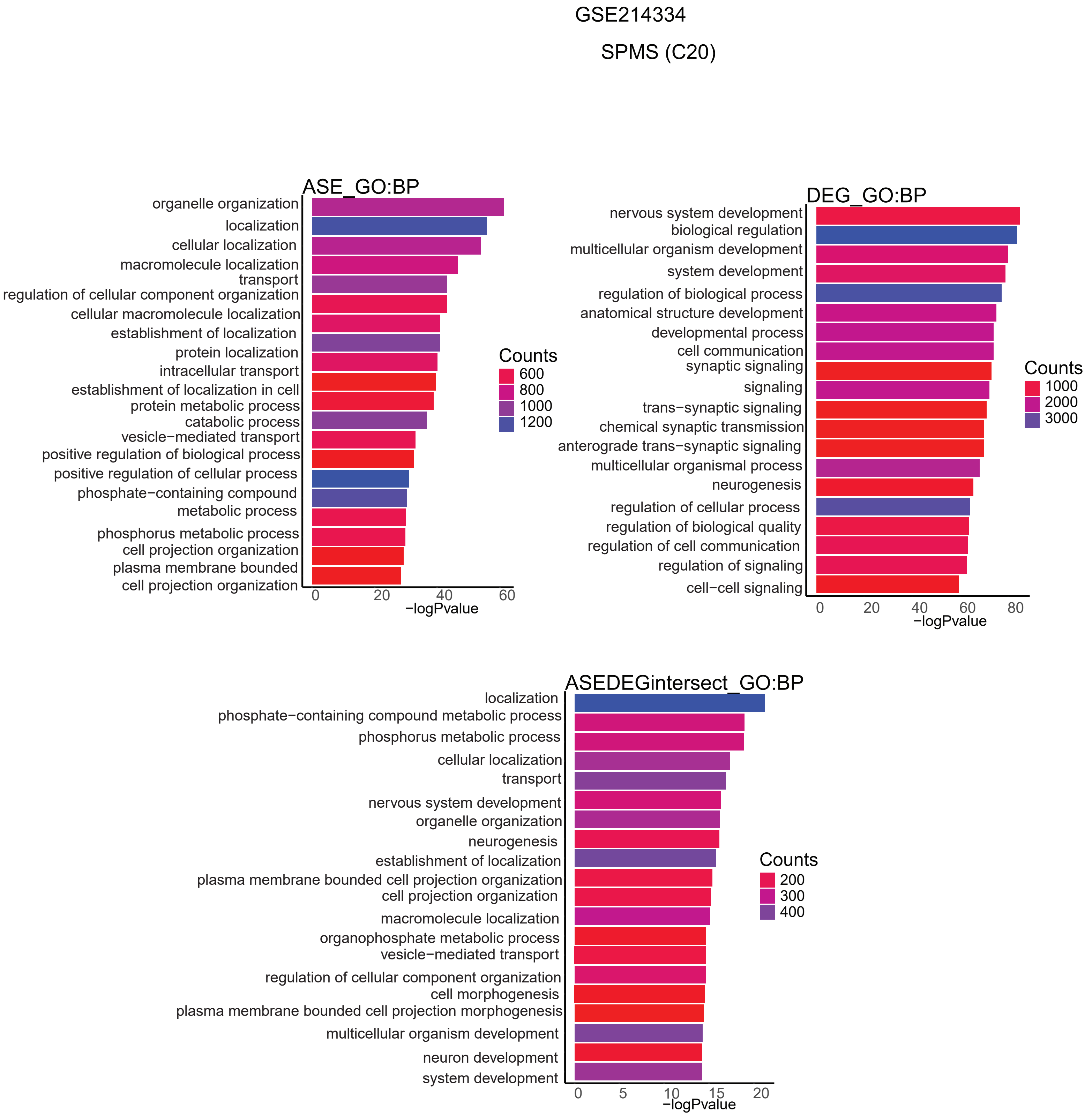
GSE214334
SPMS (C20)
ASE_GO:BP
organelle organization
localization
cellular localization
macromolecule localization
transport
regulation of cellular component organization
cellular macromolecule localization
establishment of localization
protein localization
intracellular transport
establishment of localization in cell
protein metabolic process
catabolic process
vesicle−mediated transport
positive regulation of biological process
positive regulation of cellular process
phosphate−containing compound
metabolic process
phosphorus metabolic process
cell projection organization
plasma membrane bounded
cell projection organization
0
20
40
60
−logPvalue
Counts
600
800
1000
1200
DEG_GO:BP
nervous system development
biological regulation
multicellular organism development
system development
regulation of biological process
anatomical structure development
developmental process
cell communication
synaptic signaling
signaling
trans−synaptic signaling
chemical synaptic transmission
anterograde trans−synaptic signaling
multicellular organismal process
neurogenesis
regulation of cellular process
regulation of biological quality
regulation of cell communication
regulation of signaling
cell−cell signaling
0
20
40
60
80
−logPvalue
Counts
1000
2000
3000
ASEDEGintersect_GO:BP
localization
phosphate−containing compound metabolic process
phosphorus metabolic process
cellular localization
transport
nervous system development
organelle organization
neurogenesis
establishment of localization
plasma membrane bounded cell projection organization
cell projection organization
macromolecule localization
organophosphate metabolic process
vesicle−mediated transport
regulation of cellular component organization
cell morphogenesis
plasma membrane bounded cell projection morphogenesis
multicellular organism development
neuron development
system development
0
5
10
15
20
−logPvalue
Counts
200
300
400

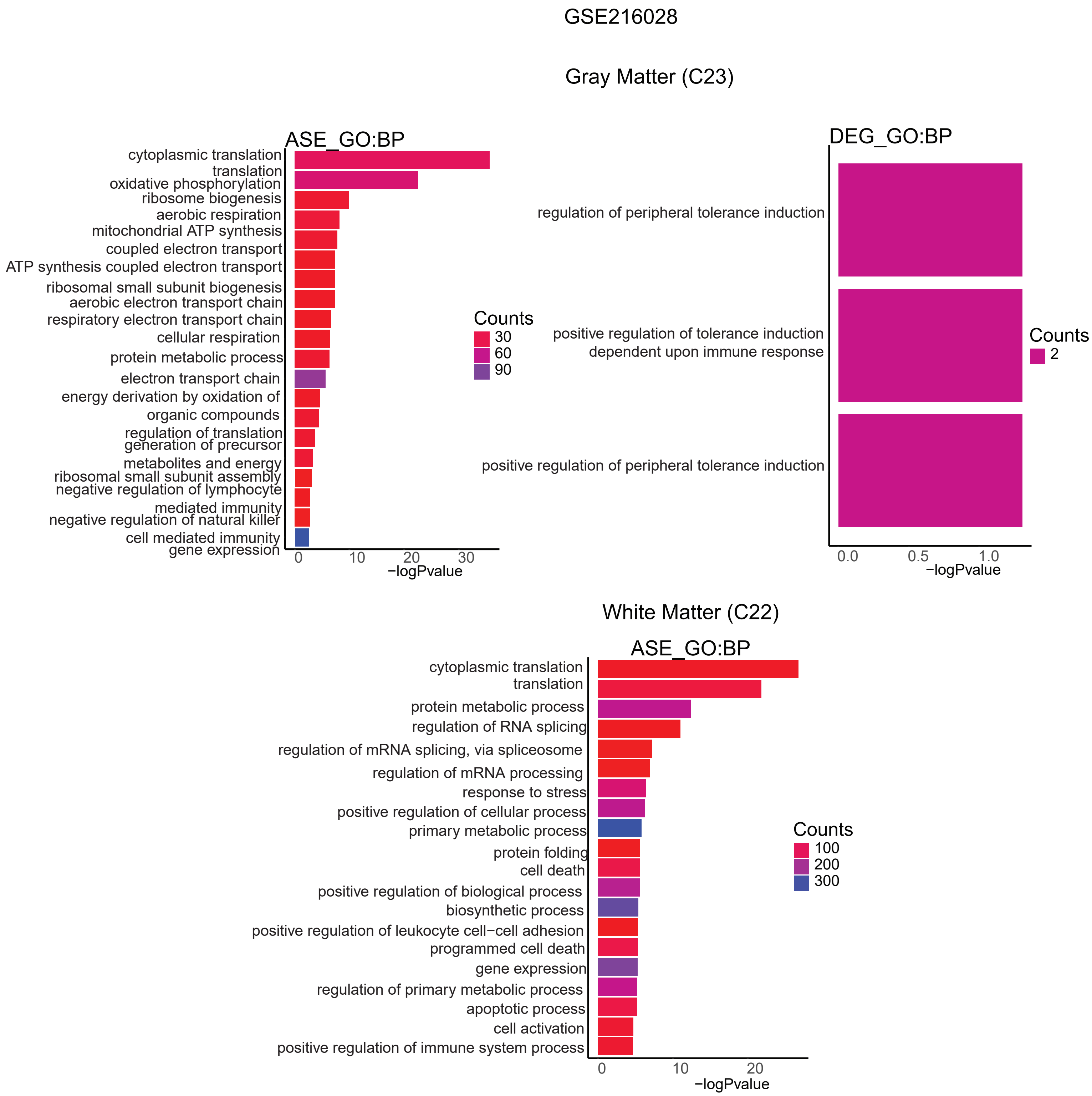
GSE216028
Gray Matter (C23)
ASE_GO:BP
cytoplasmic translation
translation
oxidative phosphorylation
ribosome biogenesis
aerobic respiration
mitochondrial ATP synthesis
coupled electron transport
ATP synthesis coupled electron transport
ribosomal small subunit biogenesis
aerobic electron transport chain
respiratory electron transport chain
cellular respiration
protein metabolic process
electron transport chain
energy derivation by oxidation of
organic compounds
regulation of translation
generation of precursor
metabolites and energy
ribosomal small subunit assembly
negative regulation of lymphocyte
mediated immunity
negative regulation of natural killer
cell mediated immunity
gene expression
0
10
20
30
−logPvalue
Counts
30
60
90
DEG_GO:BP
regulation of peripheral tolerance induction
positive regulation of tolerance induction
dependent upon immune response
positive regulation of peripheral tolerance induction
0.0
0.5
1.0
−logPvalue
Counts
2
White Matter (C22)
ASE_GO:BP
cytoplasmic translation
translation
protein metabolic process
regulation of RNA splicing
regulation of mRNA splicing, via spliceosome
regulation of mRNA processing
response to stress
positive regulation of cellular process
primary metabolic process
protein folding
cell death
positive regulation of biological process
biosynthetic process
positive regulation of leukocyte cell−cell adhesion
programmed cell death
gene expression
regulation of primary metabolic process
apoptotic process
cell activation
positive regulation of immune system process
0
10
20
−logPvalue
Counts
100
200
300
