# Supplementary material for: Systematic Analysis of Alternative Splicing in Transcriptomes of Multiple Sclerosis Patient Brain Samples
Source: Int J Mol Sci. 2025 Aug 23;26(17):8195. doi: 10.3390/ijms26178195 (PMC12428622; doi:10.3390/ijms26178195)
Supplement: Supplementary file 1 [file ijms-26-08195-s001.zip › Supplemental Tables.pdf]

Supplemental Files

Supplementary Figure S1: GO:BP Enrichment analysis for ASEs, DEGs, and intersects from all the comparisons. Counts represent the number of significant genes in category.

Supplementary Figure S2: Correlation plots for each comparison with correlation coefficients.

Supplementary Figure S3: Enriched GO:BP pathways for genes that are alternatively spliced in different brain regions; cortex, frontal cortex, occipital cortex, perietal cortex, hippocampus, choroid plexus, and internal capsule. Counts represent the number of significant genes in category.

Supplemental Table S1: Common alternatively spliced genes in PMS comparisons

Gene Symbol	Gene Description	GSE207680 (C18)	GSE214334 (C19)	GSE214334 (C20)
AARS1	Alanyl-tRNA Synthetase 1	RI	RI	RI
ACADVL	Acyl-CoA Dehydrogenase Very Long Chain	RI	RI	RI
ACTB	Actin Beta	RI	RI	RI
ADD1	Adducin 1	A5SS	SE	SE, RI, A5SS
AGTPBP1	ATP/GTP Binding Protein 1	SE,A3SS	SE	SE
AHI1	Abelson Helper Integration Site 1	SE	SE	SE
AMZ2	Archaelysin Family Metallopeptidase 2	SE	RI	SE, RI, A5SS
ANK3	Ankyrin 3	RI	A3SS	SE, A3SS
ANKRD36	Ankyrin Repeat Domain 36	SE	SE	SE
AP1G2	Adaptor Related Protein Complex 1 Gamma 2 Subunit	RI	RI	SE, RI
ATXN3	Ataxin 3	MXE	MXE	SE, MXE
BCLAF1	BCL2 Associated Transcription Factor 1	SE	A3SS	SE
CAPZB	Capping Actin Protein Of Muscle Z-Line Beta Subunit	RI	SE	SE, RI
CARS1	Cysteinyl-tRNA Synthetase 1	RI,A3SS	RI	SE, A3SS, RI, A5SS
CC2D2A	Coiled-Coil And C2 Domain Containing 2A	RI	SE	SE, RI
CCDC7	Coiled-Coil Domain Containing 7	A5SS	SE	SE, MXE
CDC42BPA	CDC42 Binding Protein Kinase Alpha	SE, MXE	SE, MXE	SE, MXE
CDC42BPB	CDC42 Binding Protein Kinase Beta	SE	SE	SE
CDK10	Cyclin Dependent Kinase 10	RI	MXE	RI, A5SS
CEP170	Centrosomal Protein 170	A5SS	RI	SE, RI
CEP290	Centrosomal Protein 290	SE	RI	RI
CERS2	Ceramide Synthase 2	RI	RI	RI
CGRRF1	Cell Growth Regulator With Ring Finger Domain 1	A5SS	A5SS	SE
CIBAR1	CIB1 Adaptor Protein	RI	SE	SE, RI, A5SS
CLN3	CLN3 Lysosomal Trafficking Regulator	RI	RI, A5SS	RI
CMC2	COX Assembly Mitochondrial Protein 2	SE	SE	SE
CSNK1D	Casein Kinase 1 Delta	RI	SE	SE
CTC1	CST Complex Subunit 1	RI	SE	RI
CTNNB1	Catenin Beta 1	RI	RI	A3SS, RI
DDR1	Discoidin Domain Receptor Tyrosine Kinase 1	SE	SE	SE
DRG2	Developmentally Regulated GTP Binding Protein 2	RI	A3SS	RI
DTNA	Dystrobrevin Alpha	SE	SE	SE, MXE
DYNC1H1	Dynein Cytoplasmic 1 Heavy Chain 1	RI	RI	RI
EDC4	Enhancer Of Decapping 4	RI	RI	RI
ELOVL5	ELOVL Fatty Acid Elongase 5	SE	SE	SE
EPB41L1	Erythrocyte Membrane Protein Band 4.1 Like 1	SE	SE, MXE	SE
EXOSC3	Exosome Component 3	SE	SE	SE
FAM193B	Family With Sequence Similarity 193 Member B	SE	SE	SE, A3SS
FIG4	FIG4 Phosphoinositide 5-Phosphatase	RI	RI	RI, A5SS
FYN	FYN Proto-Oncogene, Src Family Tyrosine Kinase	MXE	SE	SE
GAPVD1	GTPase Activating Protein And VPS9 Domains 1	SE	SE	SE, A5SS
GARS1-DT	GARS1 Divergent Transcript	RI	A3SS	SE, MXE
GUK1	Guanylate Kinase 1	RI	SE, RI	SE, A3SS, RI, A5SS
HDAC3	Histone Deacetylase 3	RI	SE	RI
HDAC9	Histone Deacetylase 9	SE	A5SS	A5SS
HERC2P3	HERC2 Pseudogene 3	RI,A3SS	MXE	SE
HM13	Histocompatibility Minor 13	A5SS	SE	SE, A5SS
HNRNPC	Heterogeneous Nuclear Ribonucleoprotein C	A5SS	SE	SE
IGHMBP2	Immunoglobulin Mu Binding Protein 2	SE	SE, MXE	SE
IP6K2	Inositol Hexakisphosphate Kinase 2	SE	SE	SE
IRAG1	Inositol 1,4,5-Trisphosphate Receptor Associated 1	SE	SE, MXE	SE
KIF2A	Kinesin Family Member 2A	RI,S5SS	SE	SE, A3SS, RI
KRBA2	KRAB-A Domain Containing 2	SE	SE	SE
LINC00320	Long Intergenic Non-Protein Coding RNA 320	SE	SE	SE
MADD	MAP Kinase Activating Death Domain	A5SS	A5SS	SE, A5SS
MAP2K2	Mitogen-Activated Protein Kinase Kinase 2	RI	SE	RI

MAP4	Microtubule-Associated Protein 4	SE	SE, MXE	SE
MBD1	Methyl-CpG Binding Domain Protein 1	SE	MXE	SE, RI
MBP	Myelin Basic Protein	RI, MXE	SE, MXE	SE, MXE, A5SS
MEG3	Maternally Expressed 3	RI	SE, RI	SE, MXE
MFSD10	Major Facilitator Superfamily Domain Containing 10	RI	SE, RI	SE, MXE
MOCS2	Molybdenum Cofactor Synthesis 2	A3SS	A3SS	SE, A3SS
MPV17	MPV17 Mitochondrial Inner Membrane Protein	MXE	MXE	SE, A3SS, RI
MRPL20	Mitochondrial Ribosomal Protein L20	RI	RI	RI
MSL3	Male-Specific Lethal 3	RI, A3SS	A3SS, RI	A3SS, RI
MTCH1	Mitochondrial Carrier 1	RI	RI	RI
MYL6	Myosin Light Chain 6	RI	SE, RI	SE, A3SS, RI
NAP1L1	Nucleosome Assembly Protein 1 Like 1	SE	SE	SE
NECAB3	N-Terminal EF-Hand Calcium Binding Protein 3	RI	RI	RI
NRXN3	Neurexin 3	SE	SE	SE
OS9	OS9 Endoplasmic Reticulum Lectin	A5SS	RI	RI
PALM2AKAP2	PALM2-AKAP2 Readthrough	SE	SE	SE
PARN	Poly(A)-Specific Ribonuclease	A5SS	SE	SE, A5SS
PCNT	Pericentrin	A5SS	SE, A5SS	SE
PICALM	Phosphatidylinositol Binding Clathrin Assembly Protein	SE	SE	SE, MXE
PKD1	Polycystic Kidney Disease 1	RI	SE, RI	SE, A3SS, RI
PLOD2	Procollagen-Lysine,2-Oxoglutarate 5-Dioxygenase 2	SE	SE	SE
PPP2R3C	Protein Phosphatase 2 Regulatory Subunit B" Gamma	MXE	MXE	MXE
PTPRE	Protein Tyrosine Phosphatase Receptor Type E	A5SS	SE	SE
PTPRF	Protein Tyrosine Phosphatase Receptor Type F	SE	SE	SE, RI
PTPRZ1	Protein Tyrosine Phosphatase Receptor Type Z1	SE, A5SS	SE, MXE	SE, MXE, A5SS
RABGAP1	RAB GTPase Activating Protein 1	A5SS	A5SS	MXE
RANBP3	RAN Binding Protein 3	SE, RI	SE	RI
RBM3	RNA Binding Motif Protein 3	A5SS	SE, A5SS	SE, A5SS
RBM39	RNA Binding Motif Protein 39	SE	SE, A3SS	SE, A3SS
REX1BD	REX1 Binding Domain	RI	A3SS, RI	A3SS
RHBDL3	Rhomboid Like 3	MXE	SE, MXE	SE, MXE
RHOT1	Ras Homolog Family Member T1	SE	SE	SE
RHOT2	Ras Homolog Family Member T2	RI	A3SS	RI
RO60	Ro60 Autoantigen	SE	SE	SE
RPL10	Ribosomal Protein L10	RI	RI	SE, RI
RPL28	Ribosomal Protein L28	RI	RI	RI
RPS16	Ribosomal Protein S16	RI	RI	RI
RPS6KB2	Ribosomal Protein S6 Kinase B2	SE	RI	SE, A5SS
SEPTIN4	Septin 4	RI	RI	RI
SEPTIN7	Septin 7	RI	SE	SE, RI
SLC6A1	Solute Carrier Family 6 Member 1	RI, MXE	A3SS, A5SS	SE, RI, A5SS
SLCO1A2	Solute Carrier Organic Anion Transporter Family Member 1A2	MXE	SE, RI	SE, RI
SORBS1	Sorbin And SH3 Domain Containing 1	SE	SE	SE, MXE
SPG7	Spastic Paraplegia 7	A3SS	SE, A3SS, RI	SE, A3SS, RI
TBL1XR1	Transducin Beta Like 1 X-Linked Receptor 1	SE	SE	SE
TCF3	Transcription Factor 3	SE, MXE	SE, MXE, A3SS	A3SS
TCF4	Transcription Factor 4	SE	SE	RI
TCF7L2	Transcription Factor 7 Like 2	MXE	A5SS	SE
THUMPD2	THUMP Domain Containing 2	SE	SE	SE
TMEM116	Transmembrane Protein 116	RI	SE	SE, RI
TMEM259	Transmembrane Protein 259	RI	MXE, RI	MXE, RI
TMEM91	Transmembrane Protein 91	RI	A3SS	A3SS, RI
TNK2	Tyrosine Kinase Non-Receptor 2	MXE, A3SS	A3SS	SE, A3SS
TSNARE1	T-SNARE Domain Containing 1	A3SS	A3SS	SE
TTC19	Tetratricopeptide Repeat Domain 19	RI, A5SS	RI	SE
TTC3	Tetratricopeptide Repeat Domain 3	SE	SE	SE, MXE
UBE2F	Ubiquitin Conjugating Enzyme E2 F	MXE	MXE	SE, MXE

UBE3A	Ubiquitin Protein Ligase E3A	SE, RI	SE, A3SS, RI	RI, A5SS
UBQLN1	Ubiquilin 1	RI	SE	RI
UBXN11	UBX Domain Protein 11	SE	RI	RI
WBP1	WW Domain Binding Protein 1	SE, A5SS	RI	SE, RI, A5SS
WDR19	WD Repeat Domain 19	MXE	SE	SE
XRRA1	X-Ray Radiation Resistance Associated 1	MXE	SE	SE
ZC3H14	Zinc Finger CCCH-Type Containing 14	A5SS	SE, MXE	SE, RI, A5SS
ZEB1	Zinc Finger E-Box Binding Homeobox 1	SE	A3SS	SE
ZMYND8	Zinc Finger MYND-Type Containing 8	A5SS	SE	SE
ZZZ3	Zinc Finger ZZ-Type Containing 3	RI	SE	SE, RI

Supplemental Table S2- Identical ASE in CC from two studies (GSE123496 and GSE111972)

Gene Symbol	chr	exonStart	exonEnd	upstreamES	upstreamEE	downstreamES	downstreamEE	ASE
A2M	chr12	9067711	9068224	9067711	9067839	9068182	9068224	RI
ACSL1	chr4	184770254	184770332	184770398	184770476	184768315	184768450	MXE
ADAM28	chr8	24353769	24353832	24351986	24352052	24354383	24354472	SE
AKAP8L	chr19	15398629	15399410	15398629	15398766	15399301	15399410	RI
AMPD3	chr11	10478525	10478730	10461514	10461740	10482062	10482225	SE
BIN1	chr2	127068162	127068255	127063932	127064018	127068923	127069031	SE
CLK1	chr2	200856890	200856985	200856681	200856811	200857717	200857884	SE
CLK1	chr2	200856890	200857884	200856890	200856985	200857717	200857884	RI
COX4I1	chr16	85804936	85805864	85804936	85805104	85805732	85805864	RI
DDX5	chr17	64501056	64501143	64500548	64500773	64502009	64502069	SE
DENND5A	chr11	9143402	9143485	9142721	9142845	9144096	9144278	SE
DNAJB2	chr2	219280577	219281771	219280577	219280687	219281717	219281771	RI
EEF1D	chr8	143581053	143581328	143581053	143581154	143581228	143581328	RI
EPB41L2	chr6	130867458	130867581	130863637	130863718	130885095	130885268	SE
FTH1	chr11	61964986	61965515	61964986	61965112	61965368	61965515	RI
HEXA	chr15	72353067	72353178	72351131	72351234	72355558	72355624	SE
HNRNPH1	chr5	179615545	179615595	179614177	179614959	179616125	179616218	SE
HNRNPH1	chr5	179617798	179618060	179617798	179617932	179617988	179618060	RI
HNRNPH3	chr10	68337857	68337996	68337198	68337333	68338502	68338642	SE
INTS6	chr13	51382028	51382123	51379461	51379572	51383328	51383461	SE
LZTS2	chr10	101003506	101004166	100999475	101002946	101005457	101005715	SE
NDUFV3	chr21	42903181	42904276	42896926	42897047	42908863	42909105	SE
PHB2	chr12	6969955	6970068	6969497	6969577	6970195	6970280	SE
QKI	chr6	163563419	163563719	163563443	163563719	163561981	163562069	A3SS
RGS2	chr1	192809457	192809535	192809142	192809181	192810165	192810267	SE
RPL10A	chr6	35468946	35469529	35468946	35469027	35469380	35469529	RI
RPL28	chr19	55386569	55388048	55386569	55386693	55387929	55388048	RI
RPS9	chr19	54206552	54207647	54207397	54207647	54206275	54206462	A3SS
SNHG1	chr11	62852810	62853115	62852810	62852839	62853074	62853115	RI
SPP1	chr4	87977661	87977850	87977058	87977097	87980045	87980126	SE
SPP1	chr4	87977661	87977859	87977058	87977097	87980045	87980126	SE
TMEM59	chr1	54047266	54047372	54043372	54043525	54052999	54053192	SE
TPM3	chr1	154169304	154169383	154156968	154157721	154170399	154170469	SE
TPP1	chr11	6617625	6618915	6617625	6617776	6618775	6618915	RI
YBX3	chr12	10701959	10704148	10701959	10702134	10704059	10704148	RI
YWHAB	chr20	44901530	44904116	44901530	44901833	44903992	44904116	RI

Supplemental Table S3: Downstream effects of the alternative splicing events in CC.

Gene name	NCBI gene ID	Gene stable ID	Exon stable ID	Pfam ID	Interacting domain	Interaction type	Visualization link
A2M	2	ENSG00000175899	ENSE00003570578	PF07677	TRUE	DDI	https://exbio.wzw.tum.de/digger/ID/exon/human/ENSE00003570578
ACSL1	2180	ENSG00000151726	ENSE00002072384	PF00501	FALSE		
BIN1	274	ENSG00000136717	ENSE00000925822	PF03114	TRUE	DDI	https://exbio.wzw.tum.de/digger/ID/exon/human/ENSE00000925822
CLK1	1195	ENSG00000013441	ENSE00003505207	PF00069	TRUE	DDI and DMI	https://exbio.wzw.tum.de/digger/ID/exon/human/ENSE00003505207
COX4I1	1327	ENSG00000131143	ENSE00001176352	PF02936	TRUE	DDI	https://exbio.wzw.tum.de/digger/ID/exon/human/ENSE00001176352
DNAJB2	3300	ENSG00000135924	ENSE00003632977	PF00226	TRUE	DDI	https://exbio.wzw.tum.de/digger/ID/exon/human/ENSE00003632977
EPB41L2	2037	ENSG00000079819	ENSE00002530352	PF05902	FALSE		
FTH1	2495	ENSG00000167996	ENSE00003566928	PF00210	TRUE	DDI	https://exbio.wzw.tum.de/digger/ID/exon/human/ENSE00003566928
HEXA	3073	ENSG00000213614	ENSE00003483570	PF00728	TRUE	DDI	https://exbio.wzw.tum.de/digger/ID/exon/human/ENSE00003483570
HNRNPH1	3187	ENSG00000169045	ENSE00003463071	PF08080	TRUE	DDI	https://exbio.wzw.tum.de/digger/ID/exon/human/ENSE00003463071
HNRNPH1	3187	ENSG00000169045	ENSE00003514844	PF00076	TRUE	DDI and DMI	https://exbio.wzw.tum.de/digger/ID/exon/human/ENSE00003514844
HNRNPH3	3189	ENSG00000096746	ENSE00003478649	PF00076	TRUE	DDI	https://exbio.wzw.tum.de/digger/ID/exon/human/ENSE00003478649
RPL10A	4736	ENSG00000198755	ENSE00003578374	PF00687	TRUE	DDI	https://exbio.wzw.tum.de/digger/ID/exon/human/ENSE00003578374
RPL28	6158	ENSG00000108107	ENSE00000699559	PF01778	TRUE	DDI	https://exbio.wzw.tum.de/digger/ID/exon/human/ENSE00000699559
RPS9	6203	ENSG00000170889	ENSE00003649985	PF01479	TRUE	DDI	https://exbio.wzw.tum.de/digg

							er/ID/exon/hu man/ENSE000 03649985
TMEM59	9528	ENSG00000116209	ENSE00003499043	PF12280	FALSE		
TPM3	7170	ENSG00000143549	ENSE00001640091	PF00261	TRUE	DDI	https://exbio.w zw.tum.de/digg er/ID/exon/hu man/ENSE000 01640091
TPP1	1200	ENSG00000166340	ENSE00003643573	PF09286	FALSE		
YWHAB	7529	ENSG00000166913	ENSE00003798043	PF00244	TRUE	DDI and DMI	https://exbio.w zw.tum.de/digg er/ID/exon/hu man/ENSE000 03798043

Supplemental Table S4- GML vs GM identical in 2 studies (GSE111972 and GSE207680)

Gene Symbol	exonStart	exonEnd	Upstream ES	Upstream EE	downstreamES	downstreamEE	ASE
AAMP	218265578	218265946	218265578	218265682	218265830	218265946	RI
ACAD8	134259607	134261179	134259607	134259745	134261043	134261179	RI
ACADVL	7220765	7221058	7220765	7220830	7220923	7221058	RI
ACTB	5529160	5529663	5529160	5529400	5529534	5529663	RI
CAPZB	19344357	19345252	19344357	19344434	19345186	19345252	RI
CARS1	3001969	3002600	3001969	3002053	3002540	3002600	RI
CD37	49335713	49337033	49335713	49335786	49336908	49337033	RI
CERS2	150966755	150967202	150966755	150966862	150967073	150967202	RI
COX7C	86619352	86619878	86619352	86619496	86620667	86620766	A5SS
DDX3X	41343736	41344128	41343736	41343822	41344029	41344128	RI
DENND4B	153930538	153931064	153930538	153930603	153930691	153931064	RI
EMC4	34228428	34228589	34227692	34227846	34229752	34230156	SE
ENGASE	79081897	79082877	79081897	79082063	79083019	79083123	A5SS
EXOSC8	37002933	37004561	37002933	37003007	37004515	37004561	RI
FASTK	151076625	151077027	151076625	151076832	151076912	151077027	RI
FBXO9	53092733	53093561	53092733	53092824	53093465	53093561	RI
HDLBP	241235120	241235594	241235120	241235255	241235489	241235594	RI
HYPK	43801517	43804427	43801517	43801569	43801710	43804427	RI
LMBR1L	49106510	49106746	49105923	49105957	49106960	49107011	SE
LMBR1L	49106544	49106746	49105923	49105957	49106960	49107045	SE
LZTR1	20994869	20995872	20994869	20995026	20995745	20995872	RI
METTL17	20994793	20995233	20994793	20994901	20995164	20995233	RI
MKNK2	2037464	2039856	2037464	2037829	2040133	2040177	A3SS
MKNK2	2039630	2039856	2037464	2037829	2040133	2040177	SE
MRPL20	1405808	1407019	1405808	1405886	1406908	1407019	RI
MS4A6A	60175401	60175611	60175505	60175611	60173027	60173129	A5SS
MYL6	56159586	56160148	56159586	56159730	56159974	56160148	RI
NDUFV1	67610380	67611207	67610380	67610570	67610994	67611207	RI
NELFCD	58993623	58994239	58993623	58993675	58994109	58994239	RI
NOL8	92298256	92298954	92298256	92298336	92298883	92298954	RI
NSUN5P1	75415591	75416191	75415591	75415778	75415980	75416191	RI
P2RX7	121167487	121167624	121165356	121165437	121175387	121175478	SE
PFKL	44319350	44320147	44319350	44319415	44320083	44320147	RI
PICALM	86023415	86023541	86022377	86022469	86026291	86026367	SE
PITPNM1	67496177	67497436	67496177	67496345	67497230	67497436	RI
RBM39	35729461	35729527	35729311	35729365	35731940	35732135	SE
RNF213	80381546	80383070	80381546	80381727	80382978	80383070	RI
RPL4	66500948	66501504	66500948	66501105	66501374	66501504	RI
RPLP1	69453646	69455287	69453646	69453721	69455169	69455287	RI
RPS16	39433521	39433761	39433521	39433569	39433664	39433761	RI
RPS2	1962496	1963256	1962496	1962909	1963148	1963256	RI
RPS28	8321655	8322091	8321655	8321703	8321952	8322091	RI
SCARB1	124782682	124786503	124782682	124782811	124786356	124786503	RI
SMARCC2	56164302	56164731	56164647	56164731	56163344	56163765	A5SS
SNHG15	44983821	44986097	44983821	44984426	44986020	44986097	RI
SRSF3	36599820	36600276	36598848	36598983	36601151	36601190	SE
SRSF5	69768797	69770540	69768797	69769251	69770466	69770540	RI
SSR3	156544293	156544439	156544307	156544439	156543003	156543269	A5SS
TTLL3	9817644	9820741	9817644	9818920	9820545	9820741	RI
UBE2B	134388976	134389052	134388324	134388413	134390224	134390321	SE
UBQLN1	83666349	83669327	83666349	83666433	83669184	83669327	RI
YWHAB	44901530	44904116	44901530	44901833	44903992	44904116	RI

Supplemental Table S5- Downstream effects of the alternative splicing events in GML

Gene name	NCBI gene ID	Gene stable ID	Exon stable ID	Pfam ID	Interacting domain	Interaction type	Visualization link
AAMP	14	ENSG00000127837	ENSE00000876140	PF00400	TRUE	DDI and DMI	https://exbio.wzw.tum.de/digger/ID/exon/human/ENSE00000876140
AAMP	14	ENSG00000127837	ENSE00000785678	PF12894	TRUE	DDI	https://exbio.wzw.tum.de/digger/ID/exon/human/ENSE00000785678
ACAD8	27034	ENSG00000151498	ENSE00003532466	PF02770	TRUE	DDI	https://exbio.wzw.tum.de/digger/ID/exon/human/ENSE00003532466
ACAD8	27034	ENSG00000151498	ENSE00003545985	PF00441	TRUE	DDI	https://exbio.wzw.tum.de/digger/ID/exon/human/ENSE00003545985
ACADVL	37	ENSG00000072778	ENSE00003678875	PF02771	TRUE	DDI	https://exbio.wzw.tum.de/digger/ID/exon/human/ENSE00003678875
ACTB	60	ENSG00000075624	ENSE00003542066	PF00022	TRUE	DDI	https://exbio.wzw.tum.de/digger/ID/exon/human/ENSE00003542066
CAPZB	832	ENSG00000077549	ENSE00003691575	PF01115	TRUE	DDI and DMI	https://exbio.wzw.tum.de/digger/ID/exon/human/ENSE00003691575
CD37	951	ENSG00000104894	ENSE00003493503	PF00335	FALSE	None	
CERS2	29956	ENSG00000143418	ENSE00003702046	PF03798	FALSE	None	
COX7C	1350	ENSG00000127184	ENSE00003492291	PF02935	TRUE	DDI	https://exbio.wzw.tum.de/digger/ID/exon/human/ENSE00003492291
DDX3X	1654	ENSG00000215301	ENSE00003721693	PF00270	TRUE	DDI	https://exbio.wzw.tum.de/digger/ID/exon/human/ENSE00003721693
EMC4	51234	ENSG00000128463	ENSE00003691355	PF06417	FALSE	None	
ENGASE	64772	ENSG00000167280	ENSE00001111730	PF03644	FALSE	None	
EXOSC8	11340	ENSG00000120699	ENSE00003515150	PF01138	TRUE	DDI	https://exbio.wzw.tum.de/digger/ID/exon/human/ENSE00003515150

FASTK	10922	ENSG00000164896	ENSE00003532150	PF08373	FALSE	None	
HDLBP	3069	ENSG00000115677	ENSE00003462881	PF00013	TRUE	DDI	https://exbio.wzw.tum.de/digger/ID/exon/human/ENSE00003462881
LZTR1	8216	ENSG00000099949	ENSE00003542765	PF00651	TRUE	DDI	https://exbio.wzw.tum.de/digger/ID/exon/human/ENSE00003542765
METTL17	64745	ENSG00000165792	ENSE00003608411	PF09243	FALSE	None	
MRPL20	55052	ENSG00000242485	ENSE00003480468	PF00453	TRUE	DDI	https://exbio.wzw.tum.de/digger/ID/exon/human/ENSE00003480468
MS4A6A	64231	ENSG00000110077	ENSE00003466261	PF04103	FALSE	None	
MYL6	4637	ENSG00000092841	ENSE00003536787	PF13405	TRUE	DDI	https://exbio.wzw.tum.de/digger/ID/exon/human/ENSE00003536787
NDUFV1	4723	ENSG00000167792	ENSE00003561301	PF01512	TRUE	DDI	https://exbio.wzw.tum.de/digger/ID/exon/human/ENSE00003561301
NDUFV1	4723	ENSG00000167792	ENSE00003571913	PF10531	TRUE	DDI	https://exbio.wzw.tum.de/digger/ID/exon/human/ENSE00003571913
NELFCD	51497	ENSG00000101158	ENSE00003731241	PF04858	FALSE	None	
P2RX7	5027	ENSG00000089041	ENSE00003524359	PF00864	FALSE	None	
PITPNM1	9600	ENSG00000110697	ENSE00003889941	PF02862	FALSE	None	
RPL4	6124	ENSG00000174444	ENSE00003461599	PF00573	TRUE	DDI	https://exbio.wzw.tum.de/digger/ID/exon/human/ENSE00003461599
RPL4	6124	ENSG00000174444	ENSE00003485616	PF14374	TRUE	DDI	https://exbio.wzw.tum.de/digger/ID/exon/human/ENSE00003485616
RPLP1	6176	ENSG00000137818	ENSE00000931693	PF00428	TRUE	DDI	https://exbio.wzw.tum.de/digger/ID/exon/human/ENSE00000931693
RPS16	6217	ENSG00000105193	ENSE00000706039	PF00380	TRUE	DDI	https://exbio.wzw.tum.de/digger/ID/exon/human/ENSE00000706039
RPS2	6187	ENSG00000140988	ENSE00003535003	PF00333	TRUE	DDI	https://exbio.wzw.tum.de/digger/ID/exo

							n/human/ENSE00003535003
RPS2	6187	ENSG00000140988	ENSE00003624369	PF03719	TRUE	DDI	https://exbio.wzw.tum.de/digger/ID/exon/human/ENSE00003624369
RPS28	6234	ENSG00000233927	ENSE00001777789	PF01200	TRUE	DDI	https://exbio.wzw.tum.de/digger/ID/exon/human/ENSE00001777789
SCARB1	949	ENSG00000073060	ENSE00003608768	PF01130	TRUE	DDI	https://exbio.wzw.tum.de/digger/ID/exon/human/ENSE00003608768
SRSF5	6430	ENSG00000100650	ENSE00003661344	PF00076	TRUE	DDI and DMI	https://exbio.wzw.tum.de/digger/ID/exon/human/ENSE00003661344
SSR3	6747	ENSG00000114850	ENSE00003550820	PF07074	FALSE	None	
TTLL3	26140	ENSG00000214021	ENSE00003550706	PF03133	FALSE	None	
UBE2B	7320	ENSG00000119048	ENSE00002074332	PF00179	TRUE	DDI and DMI	https://exbio.wzw.tum.de/digger/ID/exon/human/ENSE00002074332
YWHAB	7529	ENSG00000166913	ENSE00003798043	PF00244	TRUE	DDI and DMI	https://exbio.wzw.tum.de/digger/ID/exon/human/ENSE00003798043

Supplemental Table S6- Identical ASE in CD4/CD8 T-cells (GSE216028 in WML and GML)

Gene Symbol	exonStart_0base	exonEnd	upstreamES	upstreamEE	downstreamES	downstreamEE	ASE
AL590764.2	71108598	71108695	71108276	71108346	71109227	71109390	SE
ARGLU1	106557047	106559657	106557047	106557131	106559431	106559657	RI
ARHGEF1	41905589	41905827	41905759	41905827	41905174	41905261	A3SS
ARL6IP4	122981956	122982538	122981956	122982074	122982468	122982538	RI
ARPC2	218234351	218234397	218228737	218228850	218238663	218238850	SE
BIN2	51288107	51288188	51284715	51284787	51291590	51292344	SE
C9orf78	129834706	129834766	129833657	129833709	129835138	129835275	SE
CD37	49339329	49339413	49338699	49338936	49340250	49340466	SE
CD37	49339329	49340606	49340250	49340606	49338699	49338936	A3SS
CD96	111577501	111577549	111567522	111567647	111579026	111579234	SE
CD96	111579026	111579234	111567522	111567647	111585322	111585378	SE
CD96	111600725	111600914	111598119	111598210	111606699	111606792	SE
CD99	2717604	2717652	2714421	2714454	2719660	2719705	SE
CD99	2738199	2738256	2738202	2738256	2726259	2726373	A3SS
CDK5RAP3	47974399	47975337	47974399	47974448	47975158	47975337	RI
CENPT	67828473	67828578	67828473	67828559	67828666	67828843	A3SS
CENPT	67829764	67830548	67829764	67830088	67830389	67830548	RI
CHURC1	64926009	64926080	64923990	64924126	64932137	64934385	SE
CIRBP	1271139	1271246	1270866	1271036	1271328	1271360	SE
CIRBP	1273493	1273715	1271980	1272051	1274306	1274440	SE
COX5B	97646516	97646666	97646096	97646189	97647066	97647140	SE
CPNE1	35626043	35626803	35626043	35626381	35626566	35626803	RI
DDX5	64502161	64502549	64502161	64502223	64502438	64502549	RI
DENND2D	111197175	111197253	111195913	111196056	111197919	111197989	SE
EIF1	41689777	41689941	41688884	41689069	41690087	41690189	SE
ELOB	2771413	2772102	2771413	2771604	2771993	2772102	RI
EMP3	48327520	48327623	48326829	48326922	48329351	48329439	SE
EXOSC8	37008056	37008177	37008061	37008177	37006974	37007071	A3SS
GAS5	173865856	173866206	173865856	173865894	173866176	173866206	RI
GLIPR1	75487738	75487809	75481833	75482079	75490405	75490518	SE
GMFG	39333076	39333126	39329543	39329626	39335260	39335310	SE
GSTK1	143265260	143265296	143264547	143265092	143267616	143267733	SE
GTF3A	27434138	27434219	27430532	27430621	27434804	27435034	SE
GZMA	55105473	55105618	55102645	55102752	55108124	55108394	SE
GZMA	55107793	55107935	55102645	55102752	55108124	55108394	SE
H3-3B	75778809	75779184	75778809	75778963	75779046	75779184	RI
HLA-A	29944499	29945091	29944499	29944616	29945058	29945091	RI
HLA-A	29944499	29945091	29944499	29944616	29945062	29945091	RI
HLA-B	31356166	31356957	31356166	31356442	31356687	31356957	RI
HNRNPA1	54283078	54283234	54282799	54282874	54283811	54283967	SE
HNRNPC	21211405	21211566	21211405	21211563	21211809	21211923	A3SS
HNRNPU	244860334	244862534	244860334	244860474	244862460	244862534	RI
HSPB1	76302672	76303111	76302672	76303076	76303801	76303865	A5SS
HSPE1	197501073	197501238	197500378	197500439	197503038	197503128	SE
IL2RG	71110155	71110688	71110155	71110295	71110503	71110688	RI
IL32	3065620	3065687	3065403	3065494	3065783	3065826	SE
IL32	3065641	3065687	3065376	3065494	3065783	3065826	SE
IL32	3065780	3065826	3065783	3065826	3065311	3065494	A3SS
IL7R	35874448	35874542	35873200	35873648	35875511	35875521	SE
ILF3	10680345	10680443	10679815	10679927	10681018	10681028	SE
ISCU	108567189	108567268	108565320	108565431	108568830	108569368	SE
LCK	32279580	32279747	32279670	32279747	32276606	32276786	A3SS
LIMD2	63698798	63698938	63698798	63698818	63699027	63699069	A3SS
LIMD2	63698798	63699069	63698798	63698818	63699027	63699069	RI
MYL6	56158683	56159730	56158683	56158711	56159974	56160148	A5SS
MYL6	56159586	56159730	56158361	56158711	56159974	56160148	SE
MYL6	56159586	56160148	56159586	56159730	56159974	56160148	RI
MYL6	56159586	56160148	56159974	56160148	56158683	56158711	A3SS
MYL6	56159622	56159730	56158683	56158711	56159974	56160148	SE
NACA	56712439	56712908	56712439	56712552	56712785	56712908	RI
NDUFA11	5894674	5896575	5894674	5894854	5896452	5896575	RI
NDUFA11	5901334	5901454	5896904	5896997	5903611	5903754	SE
NDUFA3	54105596	54105703	54103113	54103188	54105933	54106011	SE

OAZ1	2271384	2271954	2271384	2271530	2271781	2271954	RI
PABPC1	100715461	100715601	100715470	100715601	100713086	100713181	A5SS
PCED1B-AS1	47206510	47206585	47205897	47206145	47209280	47209458	SE
PCED1B-AS1	47210639	47210761	47209280	47209458	47216164	47216200	SE
PCED1B-AS1	47216164	47216264	47208419	47210761	47216383	47216408	SE
PCED1B-AS1	47216164	47216434	47216383	47216434	47210639	47210761	A5SS
PFDN5	53295838	53296275	53295838	53295941	53296243	53296275	RI
PFDN5	53295838	53296467	53295838	53295941	53296429	53296467	RI
PPIA	44798747	44798814	44796680	44796793	44799246	44799277	SE
PTPN6	6960662	6960945	6960805	6960945	6960343	6960435	A3SS
RACK1	181241491	181242345	181241491	181241639	181242173	181242345	RI
RBM39	35704667	35705330	35704667	35704746	35705224	35705330	RI
RPL10	154399337	154399594	154399337	154399396	154399486	154399594	RI
RPL10A	35468798	35469027	35468798	35468873	35468946	35469027	RI
RPL13A	49490778	49491099	49490778	49490864	49491039	49491099	RI
RPL28	55386569	55388048	55386569	55386693	55387929	55388048	RI
RPL3	39313190	39314208	39313190	39313310	39314106	39314208	RI
RPL31	101002292	101002808	101002292	101002315	101002701	101002808	RI
RPL4	66500948	66501504	66500948	66501105	66501374	66501504	RI
RPL41	56116774	56116829	56116774	56116799	56117188	56117211	A5SS
RPL41	56116774	56116930	56116774	56116799	56117188	56117211	A5SS
RPLP1	69453646	69453721	69452817	69453020	69455169	69455287	SE
RPS11	49497193	49497595	49497193	49497325	49497519	49497595	RI
RPS12	132814971	132815224	132814971	132815088	132816460	132816563	A5SS
RPS15	1439960	1440253	1440025	1440253	1438806	1438892	A3SS
RPS15	1439967	1440253	1440025	1440253	1438806	1438892	A3SS
RPS2	1962496	1962909	1962496	1962656	1962735	1962909	RI
RPS20	56074059	56074506	56074059	56074159	56074380	56074506	RI
RPS28	8321655	8322091	8321655	8321703	8321952	8322091	RI
RPS3	75401639	75402446	75401639	75401733	75404019	75404207	A5SS
RPS3	75402303	75402446	75401639	75401733	75404019	75404207	SE
RPS3	75402351	75402446	75401639	75401733	75404019	75404156	SE
RPS9	54206552	54206724	54206275	54206462	54207397	54207522	SE
SKAP1	48363788	48363814	48345904	48346006	48396679	48396785	SE
SNRPN	24967931	24968082	24962113	24962209	24974310	24974434	SE
SNRPN	24974287	24974456	24974386	24974456	24967931	24968082	A3SS
SNRPN	24974310	24974456	24974386	24974456	24967931	24968082	A3SS
SPSB3	1777746	1778612	1777746	1777872	1777945	1778612	RI
SPSB3	1777945	1778612	1777945	1778048	1778133	1778612	RI
SPSB3	1778133	1778612	1778133	1778321	1778434	1778612	RI
SRRM1	24646666	24649029	24646666	24646789	24648858	24649029	RI
SYF2	25228997	25229123	25228117	25228235	25232103	25232211	SE
TPM3	154176114	154176248	154173083	154173201	154182987	154183042	SE
TPT1	45340711	45340785	45339993	45340184	45341041	45341086	SE
UQCRB	96231225	96231515	96230714	96231132	96231773	96231940	SE
VPS29	110492995	110493231	110491082	110492122	110496011	110496203	SE

Supplemental Table S7- Downstream effects of the alternative splicing events in CD4/CD8 T-cells

Gene name	NCBI gene ID	Gene stable ID	Exon stable ID	Pfam ID	Interacting domain	Interaction type	Visualization link
ARGLU1	55082	ENSG00000134884	ENSE00003502898	PF15346	FALSE		
ARHGEF1	9138	ENSG00000076928	ENSE00003543569	PF17838	TRUE	DDI	https://exbio.wzw.tum.de/digger/ID/exon/human/ENSE00003543569
ARL6IP4	51329	ENSG00000182196	ENSE00003638724	PF10500	FALSE		
ARPC2	10109	ENSG00000163466	ENSE00003596248	PF04045	TRUE	DDI	https://exbio.wzw.tum.de/digger/ID/exon/human/ENSE00003596248
CD37	951	ENSG00000104894	ENSE00003136040	PF00335	FALSE		
CD99	4267	ENSG00000002586	ENSE00003474982	PF12301	FALSE		
CDK5RAP3	80279	ENSG00000108465	ENSE00003580139	PF05600	FALSE		
CENPT	80152	ENSG00000102901	ENSE00003694282	PF15511	TRUE	DDI	https://exbio.wzw.tum.de/digger/ID/exon/human/ENSE00003694282
CENPT	80152	ENSG00000102901	ENSE00003555720	PF16171	FALSE		
CHURC1	91612	ENSG00000258289	ENSE00003544305	PF06573	FALSE		
CIRBP	1153	ENSG00000099622	ENSE00003619232	PF00076	TRUE	DDI and DMI	https://exbio.wzw.tum.de/digger/ID/exon/human/ENSE00003619232
CPNE1	8904	ENSG00000214078	ENSE00003489600	PF07002	FALSE		
DDX5	1655	ENSG00000108654	ENSE00003621662	PF00271	TRUE	DDI	https://exbio.wzw.tum.de/digger/ID/exon/human/ENSE00003621662
DENND2D	79961	ENSG00000162777	ENSE00003494377	PF02141	FALSE		
EIF1	10209	ENSG00000173812	ENSE00003487276	PF01253	TRUE	DDI	https://exbio.wzw.tum.de/digger/ID/exon/human/ENSE00003487276
EMP3	2014	ENSG00000142227	ENSE00003590409	PF00822	FALSE		
EXOSC8	11340	ENSG00000120699	ENSE00003585628	PF01138	TRUE	DDI	https://exbio.wzw.tum.de/digger/ID/exon/human/ENSE00003585628
EXOSC8	11340	ENSG00000120699	ENSE00003585628	PF03725	TRUE	DDI	https://exbio.wzw.tum.de/digger/ID/exon/human/ENSE00003585628
GLIPR1	11010	ENSG00000139278	ENSE00001742105	PF00188	FALSE		
GMFG	9535	ENSG00000130755	ENSE00003509014	PF00241	TRUE	DDI	https://exbio.wzw.tum.de/digger/ID/exon/human/ENSE00003509014
GSTK1	373156	ENSG00000197448	ENSE00003469052	PF01323	TRUE	DDI	https://exbio.wzw.tum.de/digger/ID/exon/human/ENSE00003469052
GZMA	3001	ENSG00000145649	ENSE00000971463	PF00089	TRUE	DDI	https://exbio.wzw.tum.de/digger/ID/exon/human/ENSE00000971463
H3	3021	ENSG00000132475	ENSE00000905164	PF00125	TRUE	DDI	https://exbio.wzw.tum.de/digger/ID/exon/human/ENSE00000905164
HLA	3105	ENSG00000206503	ENSE00003518731	PF06623	TRUE	DDI	https://exbio.wzw.tum.de/digger/ID/exon/human/ENSE00003518731
HLA	3106	ENSG00000234745	ENSE00001605232	PF00129	TRUE	DDI	https://exbio.wzw.tum.de/digger/ID/exon/human/ENSE00001605232
HSPB1	3315	ENSG00000106211	ENSE00000876954	PF00011	TRUE	DDI	https://exbio.wzw.tum.de/digger/ID/exon/human/ENSE00000876954
HSPE1	3336	ENSG00000115541	ENSE00003559349	PF00166	TRUE	DDI	https://exbio.wzw.tum.de/digger/ID/exon/human/ENSE00003559349
IL2RG	3561	ENSG00000147168	ENSE00003517902	PF09240	TRUE	DDI	https://exbio.wzw.tum.de/digger/ID/exon/human/ENSE00003517902

IL2RG	3561	ENSG00000147168	ENSE00001729247	PF00041	TRUE	DDI	https://exbio.wzw.tum.de/digger/ID/exon/human/ENSE00001729247
ILF3	3609	ENSG00000129351	ENSE00003584923	PF07528	TRUE	DDI	https://exbio.wzw.tum.de/digger/ID/exon/human/ENSE00003584923
ISCU	23479	ENSG00000136003	ENSE00003540589	PF01592	TRUE	DDI	https://exbio.wzw.tum.de/digger/ID/exon/human/ENSE00003540589
LCK	3932	ENSG00000182866	ENSE00001292351	PF07714	TRUE	DDI and DMI	https://exbio.wzw.tum.de/digger/ID/exon/human/ENSE00001292351
LIMD2	80774	ENSG00000136490	ENSE00003462804	PF00412	TRUE	DDI	https://exbio.wzw.tum.de/digger/ID/exon/human/ENSE00003462804
MYL6	4637	ENSG00000092841	ENSE00003536787	PF13405	TRUE	DDI	https://exbio.wzw.tum.de/digger/ID/exon/human/ENSE00003536787
OAZ1	4946	ENSG00000104904	ENSE00003708177	PF02100	TRUE	DDI	https://exbio.wzw.tum.de/digger/ID/exon/human/ENSE00003708177
PABPC1	26986	ENSG00000070756	ENSE00003491167	PF00076	TRUE	DDI	https://exbio.wzw.tum.de/digger/ID/exon/human/ENSE00003491167
PFDN5	5204	ENSG00000123349	ENSE00003570076	PF02996	TRUE	DDI	https://exbio.wzw.tum.de/digger/ID/exon/human/ENSE00003570076
RACK1	10399	ENSG00000204628	ENSE00003646461	PF00400	TRUE	DDI and DMI	https://exbio.wzw.tum.de/digger/ID/exon/human/ENSE00003646461
RBM39	9584	ENSG00000131051	ENSE00003572099	PF15519	FALSE		
RPL10	6134	ENSG00000147403	ENSE00003528800	PF00252	TRUE	DDI	https://exbio.wzw.tum.de/digger/ID/exon/human/ENSE00003528800
RPL10A	4736	ENSG00000198755	ENSE00003601153	PF00687	TRUE	DDI	https://exbio.wzw.tum.de/digger/ID/exon/human/ENSE00003601153
RPL13A	23521	ENSG00000142541	ENSE00003611819	PF00572	TRUE	DDI	https://exbio.wzw.tum.de/digger/ID/exon/human/ENSE00003611819
RPL28	6158	ENSG00000108107	ENSE00000699559	PF01778	TRUE	DDI	https://exbio.wzw.tum.de/digger/ID/exon/human/ENSE00000699559
RPL3	6122	ENSG00000100316	ENSE00003597413	PF00297	TRUE	DDI	https://exbio.wzw.tum.de/digger/ID/exon/human/ENSE00003597413
RPL31	6160	ENSG00000071082	ENSE00001665150	PF01198	TRUE	DDI	https://exbio.wzw.tum.de/digger/ID/exon/human/ENSE00001665150
RPL4	6124	ENSG00000174444	ENSE00003461599	PF00573	TRUE	DDI	https://exbio.wzw.tum.de/digger/ID/exon/human/ENSE00003461599
RPL4	6124	ENSG00000174444	ENSE00003485616	PF14374	TRUE	DDI	https://exbio.wzw.tum.de/digger/ID/exon/human/ENSE00003485616
RPL41	6171	ENSG00000229117	ENSE00002426395	PF05162	FALSE		
RPLP1	6176	ENSG00000137818	ENSE00000931693	PF00428	TRUE	DDI	https://exbio.wzw.tum.de/digger/ID/exon/human/ENSE00000931693
RPS11	6205	ENSG00000142534	ENSE00003691005	PF16205	TRUE	DDI	https://exbio.wzw.tum.de/digger/ID/exon/human/ENSE00003691005
RPS12	6206	ENSG00000112306	ENSE00001379351	PF01248	TRUE	DDI	https://exbio.wzw.tum.de/digger/ID/exon/human/ENSE00001379351
RPS15	6209	ENSG00000115268	ENSE00003597947	PF00203	TRUE	DDI	https://exbio.wzw.tum.de/digger/ID/exon/human/ENSE00003597947
RPS2	6187	ENSG00000140988	ENSE00003596226	PF00333	TRUE	DDI	https://exbio.wzw.tum.de/digger/ID/exon/human/ENSE00003596226
RPS2	6187	ENSG00000140988	ENSE00003624369	PF03719	TRUE	DDI	https://exbio.wzw.tum.de/digger/ID/exon/human/ENSE00003624369
RPS20	6224	ENSG00000008988	ENSE00000980340	PF00338	TRUE	DDI	https://exbio.wzw.tum.de/digger/ID/exon/human/ENSE00000980340
RPS28	6234	ENSG00000233927	ENSE00001777789	PF01200	TRUE	DDI	https://exbio.wzw.tum.de/digger/ID/exon/human/ENSE00001777789
RPS3	6188	ENSG00000149273	ENSE00003606545	PF07650	TRUE	DDI	https://exbio.wzw.tum.de/digger/ID/exon/human/ENSE00003606545
RPS3	6188	ENSG00000149273	ENSE00003524915	PF00189	TRUE	DDI	https://exbio.wzw.tum.de/digger/ID/exon/human/ENSE00003524915
SPSB3	90864	ENSG00000162032	ENSE00003492934	PF00622	TRUE	DDI	https://exbio.wzw.tum.de/digger/ID/exon/human/ENSE00003492934
SRRM1	10250	ENSG00000133226	ENSE00003475915	PF01480	TRUE	DDI	https://exbio.wzw.tum.de/digger/ID/exon/human/ENSE00003475915
TPM3	7170	ENSG00000143549	ENSE00003527810	PF00261	TRUE	DDI	https://exbio.wzw.tum.de/digger/ID/exon/human/ENSE00003527810

TPT1	7178	ENSG00000133112	ENSE00003458609	PF00838	TRUE	DDI	https://exbio.wzw.tum.de/digger/ID/exon/human/ENSE00003458609
VPS29	51699	ENSG00000111237	ENSE00003612453	PF12850	TRUE	DDI	https://exbio.wzw.tum.de/digger/ID/exon/human/ENSE00003612453

Supplemental Table S8 – Additional identical alternative splicing events only in CD4+ T-cells from white matter and gray matter tissues

Gene Symbol	exonStart_0base	exonEnd	upstreamES	upstreamEE	downstreamES	downstreamEE	ASE
AC040162.1	67934796	67935478	67934796	67934948	67935295	67935478	RI
ACTG1	81512231	81512781	81512231	81512360	81512733	81512781	RI
AL121845.3	63737972	63738060	63737844	63737902	63738182	63738441	SE
ATP5MF	99459146	99459263	99458189	99458355	99460085	99460193	SE
ATP5MPL	103915065	103915189	103912509	103913984	103921469	103921511	SE
CCL5	35875560	35875642	35871490	35872464	35878527	35878639	SE
CD3D	118340374	118340593	118339450	118339494	118342552	118342631	SE
CD3D	118339774	118339906	118339450	118339494	118342552	118342647	SE
CHD2	92929029	92929091	92927243	92927330	92939577	92939718	SE
CHD2	92929029	92929091	92927243	92927330	92937517	92937588	SE
HMGN1	39345829	39345958	39345145	39345274	39348291	39348339	SE
MRPL33	27774423	27774530	27772673	27772692	27779432	27779697	SE
OCIAD2	48899828	48899925	48892771	48894053	48904483	48904611	SE
PIK3IP1	31289314	31289393	31281593	31283288	31289498	31289699	SE
PSME2	24144399	24145155	24144399	24144468	24145057	24145155	RI
PTPRC	198696711	198696909	198692346	198692373	198703297	198703372	SE
RBM6	50061461	50062108	50061461	50061547	50061961	50062108	RI
REX1BD	18590853	18592336	18590853	18590933	18592107	18592336	RI
RPS25	119017924	119018053	119017361	119017545	119018281	119018330	SE
RSRC2	122522142	122522299	122518838	122521428	122526847	122526936	SE
SRP14	40038281	40038948	40038281	40038394	40038875	40038948	RI
TBC1D10C	67405902	67406017	67405906	67406017	67405594	67405701	A3SS
TECR	14563657	14563903	14563657	14563702	14563799	14563903	RI
TUBA4A	219251193	219251713	219251193	219251323	219252007	219252230	A3SS
U2SURP	143012221	143012373	143012221	143012353	143014310	143014409	A5SS
UQCRQ	132866868	132867687	132866868	132867035	132867487	132867687	RI
VIM	17235168	17236379	17235168	17235389	17235845	17236379	RI
