# Supplementary figures and images for: Systematic Analysis of Alternative Splicing in Transcriptomes of Multiple Sclerosis Patient Brain Samples
Source: Int J Mol Sci. 2025 Aug 23;26(17):8195. doi: 10.3390/ijms26178195 (PMC12428622; doi:10.3390/ijms26178195)

Figure S2

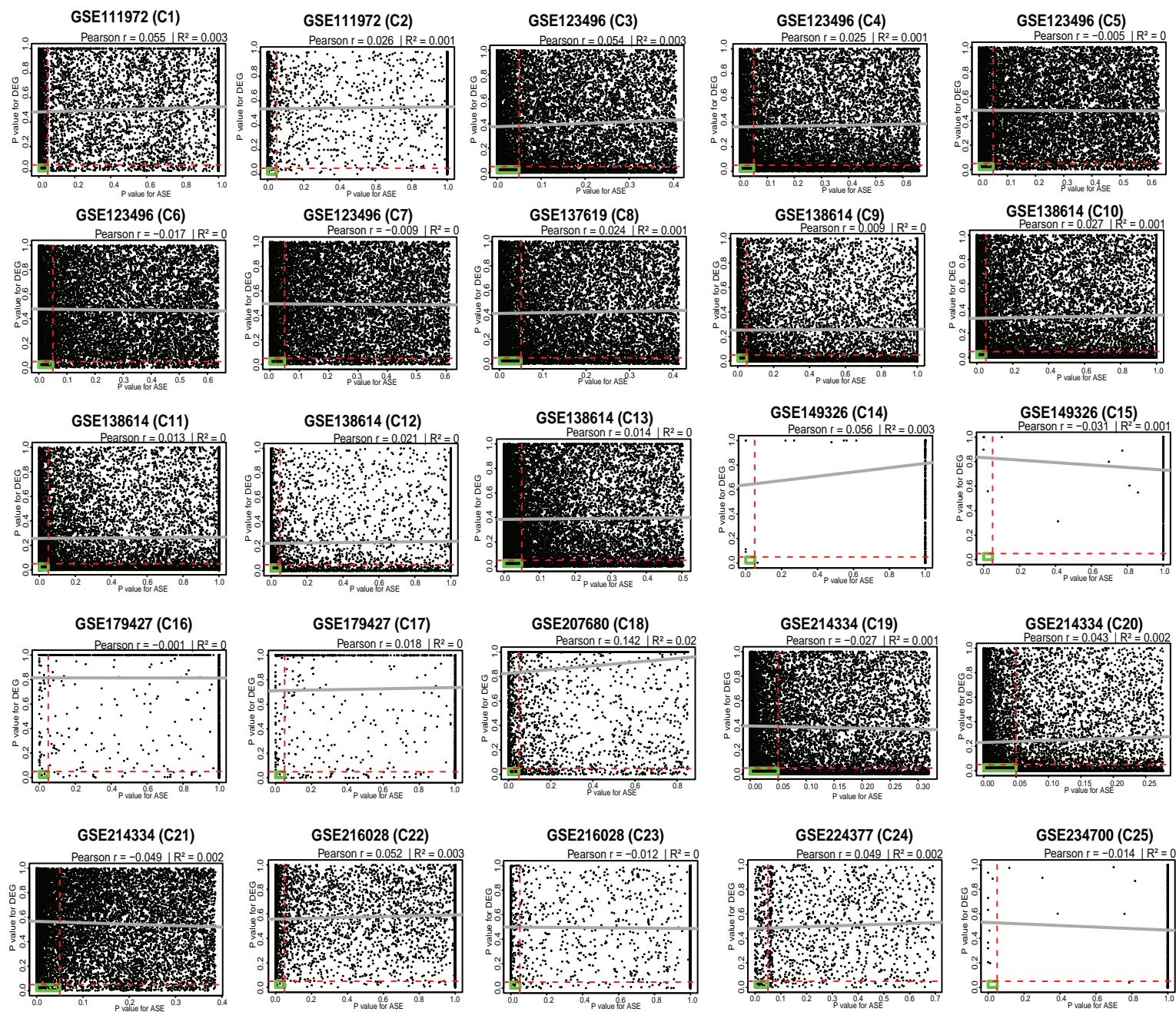

Supplement: Supplementary file 1 [file ijms-26-08195-s001.zip › Figure S2.pdf]

Figure S3

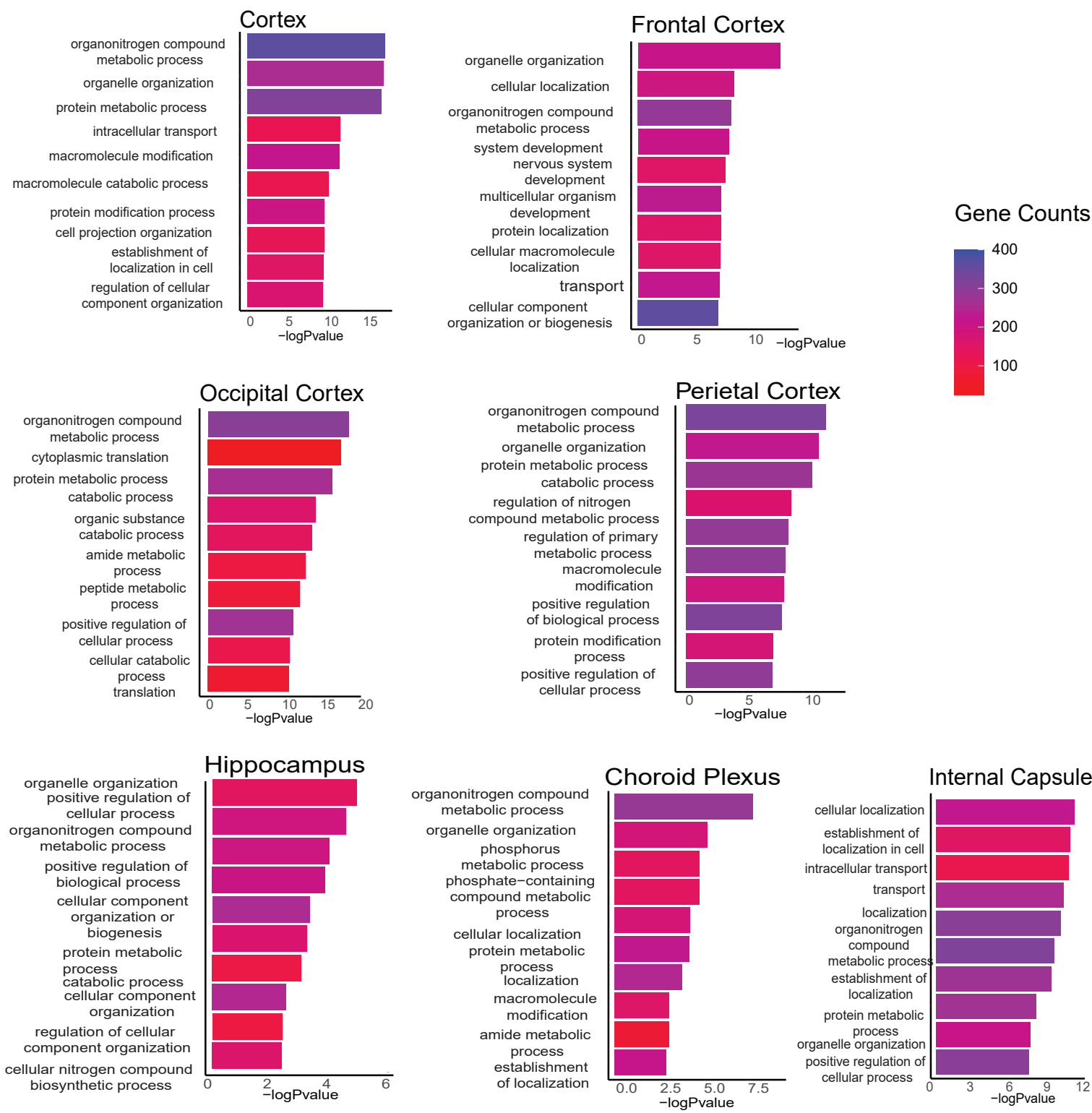

Supplement: Supplementary file 1 [file ijms-26-08195-s001.zip › Figure S3.pdf]
